# Valorisation of agricultural waste derived biochars in aquaculture to remove organic micropollutants from water – experimental study and molecular dynamics simulations

**DOI:** 10.1016/j.jenvman.2021.113717

**Published:** 2021-12-15

**Authors:** Wojciech Mrozik, Babak Minofar, Thunchanok Thongsamer, Nathacha Wiriyaphong, Sasiwimol Khawkomol, Jidapa Plaimart, John Vakros, Hrissi Karapanagioti, Soydoa Vinitnantharat, David Werner

**Affiliations:** a School of Engineering, Newcastle University, Newcastle upon Tyne, NE1 7RU, United Kingdom; b Laboratory of Structural Biology and Bioinformatics, Institute of Microbiology of the Czech Academy of Sciences, Zámek 136, 37333, Nové Hrady, Czech Republic; c Environmental Technology Program, School of Energy, Environment and Materials, King Mongkut's University of Technology Thonburi, 126 Pracha-uthit road, Bangmod, Bangkok, 10140, Thailand; d Energy and Environmental Engineering Center, Faculty of Engineering at Kamphaeng Saen, Kasetsart University, Nakhon Pathom, Thailand; e Department of Chemistry, University of Patras, Patras, 26504, Greece

**Keywords:** Biochar, Sorption, Micropollutants, Agricultural waste, Molecular dynamics simulations, Aquaculture

## Abstract

In this work, we evaluated the valorisation of agricultural waste materials by transforming coconut husks and shells, corncobs and rice straw into biochar for water treatment in aquaculture. We compared the biochars’ suitability for removal of organic micropollutants (acetaminophen, oxytetracycline, tetracycline, enrofloxacin, atrazine, diuron and diclofenac) from surface water needed for aquaculture. The biochars were prepared by three methods ranging from inexpensive drum kilns (200 °C) to pyrolysis with biogasfication (350–750 °C). Overall, antibiotics tetracycline and enrofloxacin were the most strongly sorbed micropollutants, and coconut husk biochar prepared at 750 °C was the best sorbent material. Molecular Dynamics simulations indicated that the major sorption mechanism is via π-π stacking interactions and there is a possibility of multilayer sorption for some of the micropollutants. We observed, a strong impact of ionic strength (salinity), which is an important consideration in coastal aquaculture applications. High salinity decreased the sorption for antibiotics oxytetracycline, tetracycline and enrofloxacin but increased diclofenac, atrazine and diuron sorption. We considered coconut husk biochar produced in drum kilns the most practical option for biochar applications in small-scale coastal aquacultures in South Asia. Pilot trials of canal water filtration at an aquaculture farm revealed that micropollutant sorption by coconut husk biochar under real-world conditions might be 10–500 times less than observed in the laboratory studies. Even so, biochar amendment of sand enhanced the micropollutant retention, which may facilitate subsequent biodegradation and improve the quality of brackish surface water used for food production in coastal aquaculture.

## Highlights

• Biochars (BCs) derived from agricultural waste were evaluated for water treatment.
• Four wastes, three pyrolysis methods and seven micropollutants were investigated.
• Coconut husk BC pyrolysed at 750 °C was the best micropollutant sorbent.
• Molecular dynamics simulations indicated that the major sorption mechanism is via π-π stacking interactions.
• BC amendment benefits in water filtration were demonstrated for field conditions.

## Graphical abstract

**Figure:**
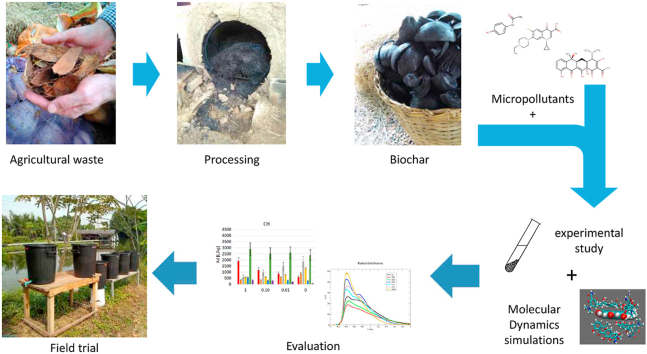

## 1 Introduction

Important for sustainable development is the application or re-use of waste or by-products from one industry (i.e. agriculture) into another industry (i.e. renewable energy production or water treatment) (COUNCIL, 2018). An example of such a waste valorisation process in the rural economy is the conversion of agricultural waste biomass into biochar by pyrolysis, for bioenergy production and/or environmental management and mitigation (Ahmad et al., 2014) (Lehmann and Joseph, 2015). In Thailand alone, coconut husks, shells and rice straw yield over 6 Mt of biomass annually (Sajjakulnukit et al., 2005). Beneficial reuse opportunities for these agricultural waste materials may discourage farmers from burning agricultural residues in the open and thus reduce significant rural air pollution problems in South Asia (Cheewaphongphan and Garivait, 2013).

In South Asia, aquaculture plays a major role in the rural economy, accounting for example for 2% of total GDP in Thailand (Sugiyama et al., 2004). However, recently the Thai aquaculture industry has suffered a major decline, which is related to problems with novel diseases and water quality (Mrozik et al., 2019). To deal with diseases and algae blooms farmers may resort to the use of antibiotics (i.e. enrofloxacin) and herbicides (i.e. diuron) (Holmström et al., 2003; Menon et al., 2020). Therefore, there is a strong need for inexpensive water purification methods, during water exchanges between the aquaculture ponds and local canals to avoid environmental impacts and cross-contamination between ponds with different management practices (i.e. intensive versus organic aquaculture). Biochar seems a perfect material for improving water quality as it is being studied as a suitable sorbent for many types of organic compounds due to its heterogeneous surface, high surface area and aromaticity. Recent studies showed its affinity for hydrophobic organic compounds (Peng et al., 2016; Zhou et al., 2010), pesticides (Mandal et al., 2017; Mohan et al., 2014; Tan et al., 2016), antibiotics (Liu et al., 2012; Yao et al., 2012) and other micropollutants (Jung et al., 2015; Rosales et al., 2017; Shan et al., 2016).

The main aim of this work was to establish the sorption properties of biochars produced from different agricultural waste materials with different methods for the removal of different organic micropollutants from water. The organic micropollutants selected for the assessment of biochar sorption properties represented a range of typical antibiotics, pesticides and common pharmaceuticals that are used in aquaculture and agriculture in South Asia or may be found in wastewater (Mrozik et al., 2019). Experimental studies were supported by molecular dynamics simulation. Finally, to study the effectiveness of biochars for water treatment in a real-life scenario, we evaluated two selected biochars as water filter medium amendment under field conditions.

## 2 Materials and methods

### 2.1 Chemicals

Acetonitrile and methanol, both LCMS grade; hydrochloric acid, formic acid and sodium hydroxide were purchased from VWR UK (Leighton Buzzard, UK). Acetaminophen (ACM), oxytetracycline (OTC), tetracycline (TC), enrofloxacin (ENFL), atrazine (ATR), diuron (DRN) and diclofenac (DIC) were bought from Sigma Aldrich (Irvine, UK). Table 1 lists the properties of the selected micropollutants.

**Table 1:** Properties of the selected pollutants.

Name	Structure	Molecular mass [g/mol]	pKa^a^	logP^a^
acetaminophen		151	9.5	0.51
atrazine		215	1.68	2.61
diclofenac		296	4.15	4.51
enrofloxacin		359	pKa_1_ : 5.88–6.06 pKa_2_: 7.70–7.74	4.7
diuron		233	n/a	2.87
tetracycline		444	multiple	0.09
oxytetracycline		460	multiple	- 0.9

a DrugBank database (DrugBank.ca, 2020).

### 2.2 Biochar preparation and characterization

Four feedstocks, namely coconut shells (CS), coconut husks (CH), rice straw (RS) and corncobs (CC), sourced in central Thailand, were transformed into biochar by the community drum kiln method (CH/CS/CC/RS) and in a pyrolysis reactor (CHP). Besides, CH, CS, and RS biomass were also transformed into biochar by pyrolysis at a fixed temperature of 550 °C (CHE550/CSE550/RSE550), and 750 °C (CHE750/CSE750). Details of the procedures are included as **Supplementary Materials**. The CH was also impregnated with chitosan (CHCHI) to increase the positive charge on its surface. It was prepared by mixing CH with chitosan solution (100 g of chitosan powder (Biolife ELAND corp. Thailand) in 10 L of 1% acetic acid) at the ratio of CH:chitosan of 5:1 by weight. Then, CHCHI was recovered by passing the solution through a fine net (>0.250 mm) and was air-dried before use.

Additional, specific physio-chemical properties of biochars are presented in **Supplementary Materials (**Table S1 **and** Table S2**)**.

### 2.3 Sorption studies

Batch sorption tests were performed in triplicate (OECD and OECD, 2000) for the chosen mixture of micropollutants (Table 1). The working solution was prepared from individual stock solutions of all compounds (1 g/L in MeOH). To achieve sample homogeneity and minimize biochar particle size-related kinetic sorption effects (Ulrich et al., 2015), the biochar was finely ground for these batch experiments. A selected amount of each biochar (in the range: 5, 10, 25, 50, 100 and 250 mg) was added to the test vials. Next, 50 mL of the aqueous solution containing each pharmaceutical at 1 mg/L was introduced. The amber glass test tubes (60 mL) were placed on a shaker to reach equilibrium sorption time at room temperature. Blank samples (without analytes) and control samples (without biochars) were treated with the same conditions. Next, the samples were filtered through syringe filters (PVDF, 0.22 μm) into chromatographic vials. Samples were then analyzed by a UHPLC/UV technique (**detailed in Supplementary Materials**). The pH experiments were adjusted with hydrochloric acid or sodium hydroxide to reach the range of 4–11. Ionic strength experiments were set for concentrations of 0, 0.01, 0.1 of 1 M sodium chloride.

### 2.4 Molecular dynamics simulations

To study the interactions between organic micropollutants and the surface of biochars, classical molecular dynamics (MD) simulations were applied (Borthakur et al., 2021). Different models of biochar and different conditions were used in simulated systems to investigate the influence of added salt and chitosan molecules on sorption. The preparation process alters the BC properties (i.e. surface area or the number of functional groups) and results in much higher aromaticity in biochars made at higher temperatures (Han et al., 2017). Therefore, we used models with 18 (for biochars <350 °C) and 34 (>500 °C) benzene rings to reflect that impact (Yerizam et al., 2013). These structures were used for CS, CH and CC biochars as general models named R18 and R34, respectively. As the RS biochars had higher sulfur content we used amended models that reflected that property (R18/34S). The chemical structures of these models are presented in Figure S5 and S6 in Supplementary Materials.

### 2.5 Filtration field trial

Field trial water filters consisted of 50 L plastic buckets with an outlet valve at the side near the bottom. The filling comprised a 7 kg layer of gravel (grain size - 2 mm), below a thin layer of sand (4 kg) followed by 11 kg of sand mixed with biochar (11 kg ∼ 10:1 w/w) and finally another layer of gravel (2 kg). The control filter was filled with sand instead of the sand/biochar mixture. The container was filled with 25 L of canal water and left to equilibrate for 15 min. Next, the flow was set to 1 L/min and individual samples (1 L) were collected up to a total flow volume of 24 L. There were two control filters with sand, two filters with CH biochar amended sand, and two filters with CHCHI biochar amended sand. The concentrations of the samples were analyzed by LC/MS/MS method as described in our earlier work (Mrozik et al., 2019). The set-up is presented in Figure S7 in Supplementary Materials.

### 2.6 Modelling and statistics

Hierarchical clustering was performed to analyze data similarities using average values of K_bc_s for all compounds for every biochar. Statistical calculations were done with Minitab 18 software. The breakthrough curves from the filtration field trial were interpreted with a numerical model which considered the sorption-retarded transport of the micropollutants in a porous medium consisting of sand for the control filters, or sand and porous biochar for the other two filters (Trinh et al., 2017; Ulrich et al., 2015). More information and the underpinning equations are provided **in Supplementary Materials**.

## 3 Results and discussion

### 3.1 Adsorption strength

**Fig. 1:**
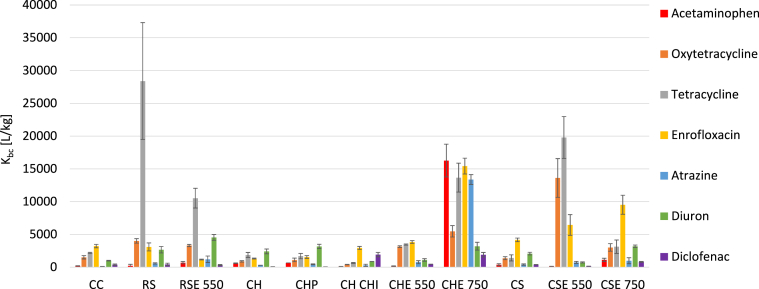
Sorption coefficients (Kbc) of micropollutants for selected biochars - initial biochar loading of 200 mg/L.

The distinction in material source and preparation methods played a crucial role in sorption properties (Table S2 in Supplementary Materials). Initial experiments compared 24 h with 7 days contact periods (Table S3 in Supplementary Materials), and the results showed only a minor increase in the percentage of adsorption for the 7 days equilibration of between 0.2 and 1.7 per cent, therefore, 24 h contact was chosen. Fig. 1 presents the linear sorption coefficients K_bc_s from the screening study with all the micropollutants and biochar types. The best universal sorption properties were observed for biochar produced from coconut husks by pyrolysis at a high, fixed temperature of 750 °C (CHE750) – 100% removal of all compounds for the two biggest loadings (Table S3 in **Supplementary Materials**). Even acetaminophen that was only weakly adsorbed on the other biochars, was strongly bound to the CHE750. The next best sorbents were CSE550 and CSE750, especially for higher biochar loadings. Rice straw biochars also exhibited relatively strong sorption, especially for oxytetracycline and tetracycline (for RS produced by the drum kiln method), and the strongest for diuron (RS550). An interesting case was CHCHI since the modification of its surface reversed the CH sorbent properties. That resulted in weaker sorption of acetaminophen, oxytetracycline, tetracycline, and atrazine, but stronger sorption of enrofloxacin, diuron and diclofenac, as compared to the unmodified biochar. Overall, the most weakly bound micropollutants were acetaminophen, atrazine and diclofenac, regardless of biochar type and loading which agrees with other studies (Caban et al., 2020).

**Fig. 2:**
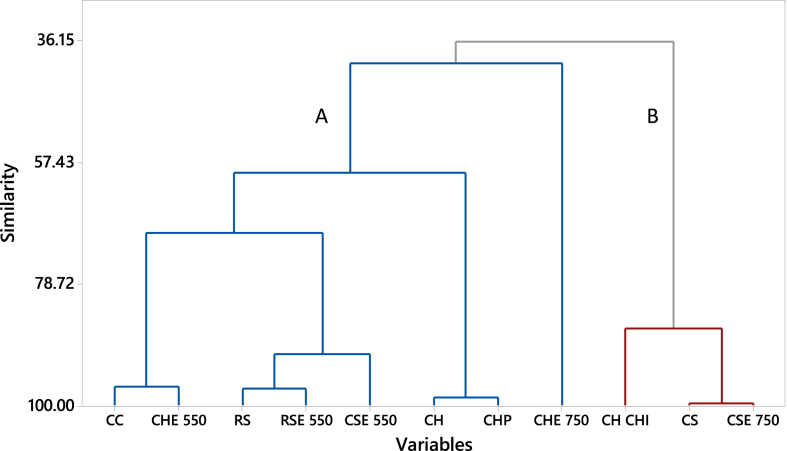
Hierarchical clustering of sorption similarity according to biochar type.

Fig. 2 shows the hierarchical clustering of similarities in sorbent properties for the full range of K_bc_s (for all compounds per sorbent). There are two main clusters, showing how both the source material and temperature of production were influential, meaning that biochars from the same source materials were found in different clusters. The common similarity of the biochars in cluster B - CS/CSE750 - was a high affinity to enrofloxacin and a moderate affinity to the other compounds. In the case of CHCHI, it was the most distinct sorption material to others. These observations are in agreement with the trends showed in Fig. 1. In Cluster A, there was a distinct separation of the most adsorbing material (CHE750) from the other biochars. The “A” subclusters: CH/CHP, RS/RSE550 and CC/CHE550 were clustered together as they showed similar sorption patterns (see Fig. 1).

**Fig. 3:**
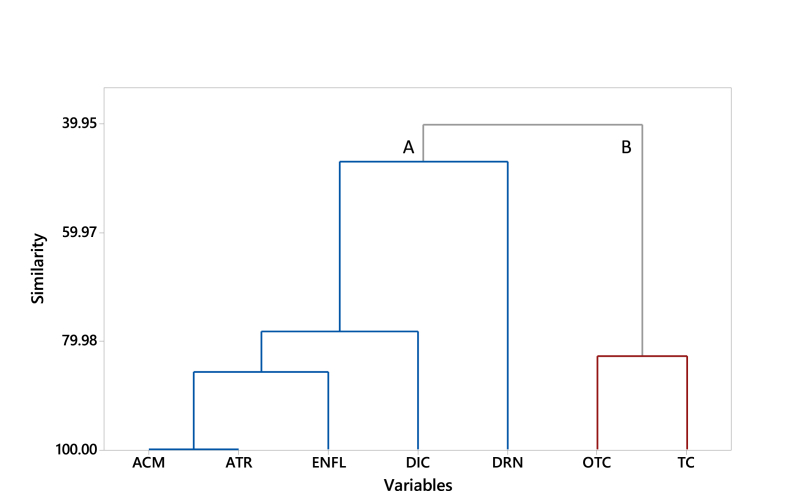
Hierarchical clustering of sorption similarity according to micropollutant types.

We also visualized the way pollutant properties affected sorption by the different biochar types. The dendrogram depicting the clustering of selected compounds according to their K_bc_ values is shown in Fig. 3. We observed a distinct separation of oxytetracycline and tetracycline (cluster B) from the rest of the micropollutants (see Fig. 1). Separation of the rest of the micropollutants was less distinct, and they formed a second cluster with three subclusters (cluster A), with the lowest similarity of diuron and enrofloxacin to the rest, perhaps due to distinction in sorption to the CHCHI. We noted the highest similarity for acetaminophen and atrazine, which was quite interesting considering their differences in charge or pK_a_. According to previous studies (Rosales et al., 2017), π-π electron donor-acceptor (EDA) interactions were a major sorption mechanism. This phenomenon is relevant for compounds with an aromatic ring in their structure as they may donate π electrons to π-electron acceptors on the biochar surface (Xie et al., 2014). Other studies (Jia et al., 2013; Shan et al., 2016) proposed such interactions as the primary mechanism for the sorption of tetracycline or oxytetracycline. However, strong electron-withdrawing groups on diclofenac or acetaminophen may have reduced π-electron availability in their aromatic rings for these types of interactions (Ji et al., 2010; Jung et al., 2015), resulting in lower sorption which is the agreement with our observations. These observations are later discussed in the molecular dynamic simulation (Section 3.4).

The competition between different organic molecules for the same sorption sites may influence the adsorption mechanism resulting usually in a decreased sorption (Jung et al., 2015; Zhang et al., 2016). In our study, a mixture of seven compounds was used, because many micropollutants will typically be simultaneously present in real-world applications. Therefore, experiments with the mixture reflect more real-life conditions than separate studies for each compound alone.

### 3.2 The effect of ionic strength and pH

Aquacultures in the coastal region of Thailand depend on brackish water. This is due to saline water intrusion with high tides up to several kilometres inland. Therefore, it was critical to assess the influence of ionic strength (IS) on the sorption properties of biochars, when considering the biochar application for water treatment in coastal aquaculture.

**Fig. 4:**
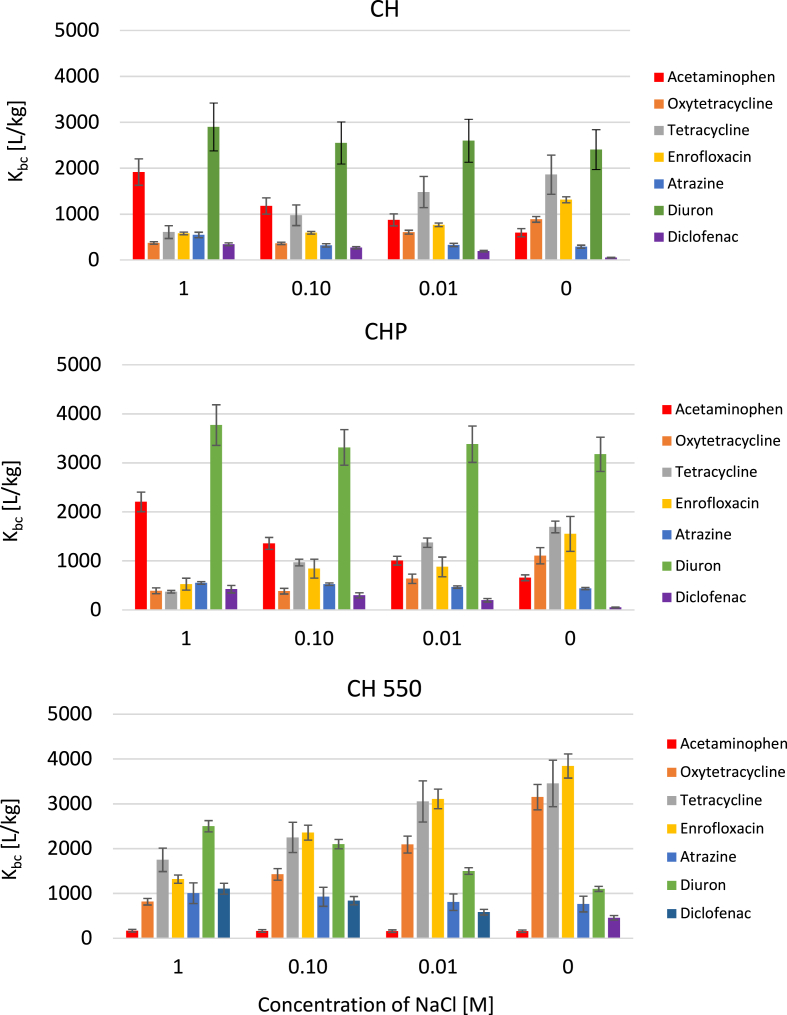
Influence of ionic strength on sorption of micropollutants on selected biochars. CH - drum kiln method; CHP - pyrolysis method; CHE 550 - pyrolysis at a fixed temperature of 550 °C.

The influence of ionic strength strongly depends on the studied system and biochar properties (Chen et al., 2015; Rosales et al., 2017). With increasing inorganic salt concentration, various phenomena occur, and thus it is not unusual to find studies that are contradictory to each other. Nonetheless, in our study, we noted a decrease in sorption with rising salinity for oxytetracycline, tetracycline and enrofloxacin (Fig. 4). This is a common observation for biochar systems and often explained by the competition of dissolved ions with ionic pollutants for adsorption sites (Chen et al., 2015; Huang et al., 2017; Inyang et al., 2015; Jia et al., 2013; Ma et al., 2021). However, other studies reported either minimal influence of the ionic strength for tetracycline (Xu et al., 2020) or even higher sorption of micropollutants in the greater presence of Na^+^ ions (Xie et al., 2014). The latter is due to the screening of the surface electrostatic charge by counter-ions, which may then lead to a higher uptake of ionizable micropollutants (Zhang et al., 2016). Another driver is the “salting out” effect that leads to a decrease in the solubility of hydrophobic organic compounds and enhanced sorption (Schwarzenbach et al., 2003). Such behavior was reported for atrazine (Ureña-Amate et al., 2005), and in our biochar systems, observations for acetaminophen, atrazine, diclofenac and diuron are in agreement with it. The higher hydrophobicity of these compounds, in comparison to oxytetracycline and tetracycline, likely supported this behavior.

As the surface charge and ionization state of the compounds often depends on the pH of the solution, pH thus influences the adsorption processes. Similarly to the influence of ionic strength, the influence of pH has been reported to range from very strong (Jia et al., 2013) to minimal (Liu et al., 2012). In our experiments, the basic pH for biochar ranged from 9.5 to 10.3 except for CHCHI that was 5. That meant that most of the compounds, in the solution, were neutral (atrazine and diuron) or anionic species (all the rest). To assess the pH influence we carried out experiments over an initial pH range from 4 to 11. For all the compounds we noticed only minor changes in the sorption. This can be explained by the high buffering properties of the biochars as the final pH values were close to the unaltered solution pH (i.e. ranging from 7 to 10.3) (Liu et al., 2012). Our results agreed with some studies i.e. for tetracycline (Shan et al., 2016) or atrazine (Zhang et al., 2015) or diclofenac (Filipinas et al., 2021), but there are also contrary results, i.e. for the removal of atrazine (Amde et al., 2015). However, it must be noted that the typical pH of canal water in Thailand's coastal aquaculture region oscillates in the range of 7.2–8.8 (Mrozik et al., 2019). That, in combination with the buffering properties of the biochars, make pH-dependent sorption less relevant.

### 3.3 Adsorption isotherms

For selected biochars, we fit data obtained in the batch sorption study with a range of pollutant concentrations to linear, BET, Langmuir and Freundlich sorption models. Isotherms were fitted if at least five data points were available and if these data points spanned at least a factor of 5 in the aqueous concentration range. Table S6 lists isotherm parameters for the selected biochars. In most cases, we observed good fits to the BET, Freundlich and Langmuir models, indicating non-linear sorption. In the literature, good fits to Langmuir have been reported for tetracycline (Jang and Kan, 2019), enrofloxacin (Zhao et al., 2019) or atrazine (Penn et al., 2018). Nonetheless, the fits to the Freundlich isotherm were often of similar quality, and such behavior was confirmed in terms of atrazine sorption on soils (Yue et al., 2017). Other studies concerning enrofloxacin (i.e. based on the structurally similar ciprofloxacin) confirmed the fit to the Freundlich model (Afzal et al., 2018; Álvarez-Esmorís et al., 2020; Hu et al., 2019; Kim et al., 2020). In our study, a few 1/n Freundlich exponent values exceeded the value of 1, showing multilayer sorption, which we then also confirmed by good fits obtained for the BET isotherm model.

### 3.4 Molecular dynamics simulations

**Fig. 5:**
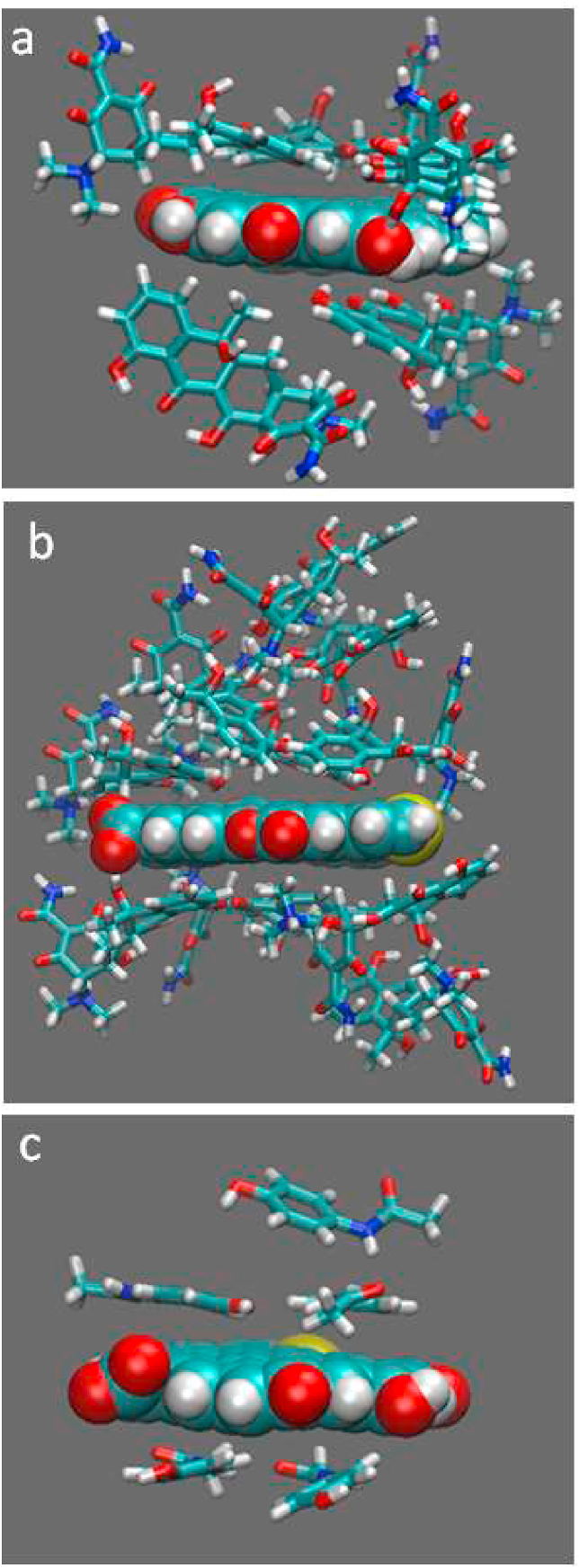
Shanshot from MD simulation showing the π-π stacking interactions of R18S biochar surface with a) hydrophobic surface of TC; b) TC where multilayer is formed c) ACM where not multilayer is formed.

**Fig. 6:**
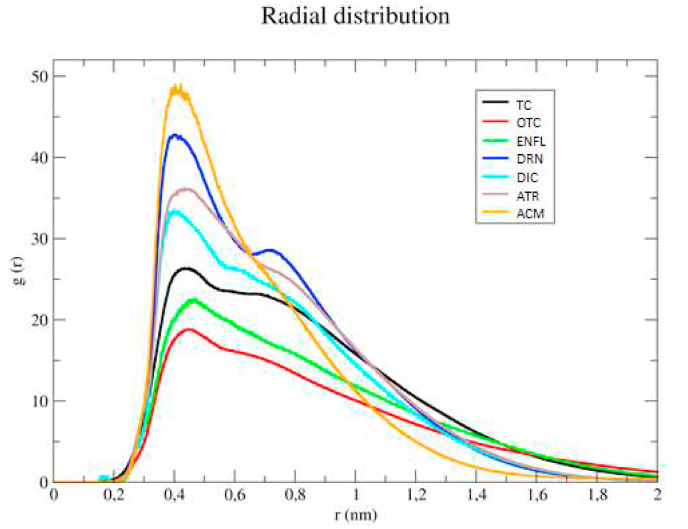
Radial distribution function of organic micro pollutant molecules center of, mass around R18 biochar molecules.

The radial distribution function (RDF) of the R18 model shows that all organic micropollutants peak at around 0.5 nm which indicates that the main interactions were of a weak hydrophobic type (Figure S7 (a-g) in Supplementary Materials). Generally, the bigger the surface area of biochar the stronger the adsorption of micropollutants which agrees with the experimental findings. However, despite this qualitative observation, the simulations results were not fully in quantitative agreement. For instance, the MD results showed that TC was less adsorbed on the surface of R34 than R18 and R18S, contrary to experimental data. Yet, we must emphasize that the main aim of modelling was to support experimental data and as a compliment to reveal the nature of interaction and mechanism of interaction. Nonetheless, the simulations revealed that the major interactions involved in sorption were hydrophobic van der Waals forces and π-π stacking interactions but there is also an occurrence of less dominant weak interactions such as hydrogen bonding. Fig. 5 (a) shows that tetracycline not only interacts via π-π stacking with the hydrophobic surface of biochar (as a major force) but also with the hydroxyl group of biochar via hydrogen bonding. Moreover, as oxytetracycline and tetracycline have bigger hydrophobic surface areas, compared to the rest of organic micropollutants, the π-π stacking between these molecules with the sorbent surface is stronger, which indicates that π-π stacking energy is size-dependent. Besides the strong π-π stacking between oxytetracycline–oxytetracycline or tetracycline-tetracycline molecules in the solution, causes clustering of micropollutants that results in multilayer sorption at the surface of biochar (Fig. 5 (b)). This phenomenon was already indicated by the Freundlich isotherm parameters. While common for oxytetracycline and tetracycline solutions, for another micropollutant like acetaminophen, such a multilayer is hardly formed on the biochar surface due to weak π-π stacking among acetaminophen molecules. In theory, acetaminophen can be adsorbed as a multilayer but due to very fast desorption, that phenomenon is not occurring (Fig. 5 (c)). Further analysis of the trajectory of MD simulations confirmed that in oxytetracycline and tetracycline systems all solvated molecules could be adsorbed at the surface of biochar as monolayer or multilayers, but such a phenomenon is not true for acetaminophen where the monolayer is dominant. In addition, due to the smaller size of acetaminophen, more molecules could be adsorbed per unit of biochar which gives a higher RDF peak intensity for acetaminophen than for oxytetracycline and tetracycline. By careful investigation of peaks of the RDF, it can be concluded that all micropollutants have the highest intensity of interaction around 0.4–0.5 nm which is in agreement with typical π-π stacking interaction in molecules (Fig. 6). Peaks decline steeper for molecules, which have a smaller hydrophobic surface such as acetaminophen or diclofenac (Jung et al., 2015) than those of oxytetracycline and tetracycline, which decrease with a lesser slope due to the formation of multilayers.

The MD simulations were also used to investigate the influence of ionic strength on sorption. The decrease in adsorption is due to the competition of dissolved ions with ionic pollutants for adsorption sites (Inyang et al., 2015) which means that the ions have a propensity to the hydrophobic surface of biochar. The propensity of soft and polarizable ions such as chloride, bromide and iodide to the hydrophobic surface of the water (air/water interface) has been previously reported (Tůma et al., 2005; Vrbka et al., 2004) where non-polarizable ions such as fluoride and sodium were repelled from the interface. By knowing the propensity of polarizable ions to the hydrophobic surface it can be concluded that chloride ions can be adsorbed at the hydrophobic surface of biochar leaving fewer vacant sites available for organic micropollutants. In our simulations, the propensity of chloride ions to the biochar surface is negligible due to the small surface area. This is due to the use of diluted biochar solutions namely one molecule of biochar in each simulation box.

The simulation showed a decreased removal for oxytetracycline, tetracycline and enrofloxacin which is in agreement with the experimental observations (Figure S8 in Supplementary Materials). However, RDFs also indicated a decrease for diclofenac which was not observed in the experiments (increase). MD simulations indicated that enrofloxacin and diclofenac should have much lower adsorption at the surface of biochar because the COO^−^ moiety of these anions is ion paired with sodium ions therefore they lose their ability to make hydrogen bonds with other molecules. It is not yet clear why this trend was true for enrofloxacin and not for diclofenac in our experiments, and further research is required. Another effect highlighted by the addition of the salt is that the adsorption of smaller molecules such as acetaminophen, atrazine and diuron is increased dramatically. Na^+^ cations can make contact ion pairs with the COO^−^ groups of biochar, whereas Cl^−^ anions due to the big anion size and hydrophobic character can show a surface propensity to the hydrophobic surface of biochar. In other words, cations and ions of salt partially occupy the free adsorption sites of the biochar surface and therefore smaller micropollutants (acetaminophen, atrazine and diuron) have a higher chance to be adsorbed at the remaining available surface in comparison to bigger molecules (tetracycline, oxytetracycline).

### 3.5 Field studies and modelling

To complement the batch study data, we also tested the performance of two selected biochars in a more real-life scenario of micropollutant removal at native concentrations from brackish canal water used in coastal aquaculture downstream of Bangkok, Thailand. For this pilot-scale study, we selected CH, as it was a readily available local agricultural waste that would be within reach of the local aquaculture farmers. For comparison, we also evaluated its modified, chitosan-impregnated version (CHCHI) because of its distinct sorbent properties. During fieldwork at a family-owned case study aquaculture farm in January 2018 (Mrozik et al., 2019), we placed a set of filters alongside one canal and studied their performance in terms of the retention of micropollutants under realistic conditions. In a real-world application, a high amount of interfering compounds such as natural organic substances, non-ideal flow and non-equilibrium conditions may all contribute to reduced retention of micropollutants by biochar in a water filtration system in comparison with predictions from batch sorption studies.

**Fig. 7:**
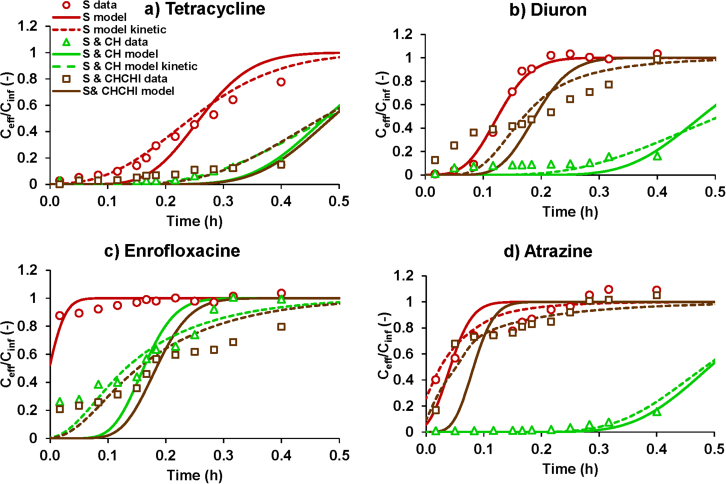
Measured breakthrough curves for the field trails and simulations with the instaneous local sorption equilibrium model (solid line) and kinetic sorption model (broken line) for a) tetracycline, b) diuron, c) enrofloxacin, and, d) atrazine S = sand; CH = coconut husk biochar; CHCHI = coconut husk biochar impregnated with chitosan.

The biochar amended sand filters showed improved performance in the retention of micropollutants as compared to the sand only control filters, which often showed almost immediate breakthroughs of the tested compounds (Fig. 7). The filters were conditioned with canal water for 15 min before the beginning of the breakthrough experiment, which then implied that breakthrough could occur instantaneously if there was no pollutant retention by the filter medium (i.e. as was observed for enrofloxacin and atrazine in the control with sand). The observations that effluent concentrations in the control (sand) tended towards the influent concentrations by the end of the monitoring period supported the modelling assumption that biodegradation had only limited effects on the breakthrough curves for the conditions of this case study with a high filtration rate and a short duration. In many cases, fronting and tailing were observed when comparing experimental data with simulated breakthrough curves based on the local sorption equilibrium assumption (Fig. 7, solid lines). The kinetic sorption model (Werner et al., 2012) could then match the data more closely (Fig. 7, broken lines). However, fronting and tailing in the breakthrough curves may also be explained by preferential flow in a porous medium, and it is difficult to derive the exact mechanisms causing these phenomena from the available field trial data. Despite these complexities, some notable trends were evident regardless of the model used for the data interpretation. We observed the best retention in the CH biochar amended filter, in which all compounds were retained except for enrofloxacin. In the CHCHI filter, we observed stronger interaction with tetracycline and enrofloxacin, but weaker interaction with atrazine, in agreement with the trends observed in the batch sorption studies.

Biochar sorption coefficients K_bc_ obtained from the field filtration experiments were about 10–500 times lower than those obtained from the batch experiments (Table 2). This is due to matrix effects such as competition for sorption sites between various constituents of the surface water matrix (i.e. organic matter), sorbent fouling and non-ideal flow conditions in the filters. For practical applications, the K_bc_ derived from the field test will provide a more realistic assessment of the biochar performance in real-world applications than the batch study data. Even though the micropollutant sorption by biochar was greatly reduced in the pilot-scale evaluation, the evidence that biochar enhances the micropollutant retention in the filter bed is encouraging. This observation is significant because aquaculture farmers will only periodically top up their ponds with canal water to compensate for evaporative water losses, mainly during the dry season (Mrozik et al., 2019). Retention of micropollutants in the filter bed during these sporadic loadings with canal water then provides the opportunity for subsequent micropollutant biodegradation in the filter bed during the much longer interim periods when there is no exchange of water between the canal and the pond (de Castro et al., 2018). The pilot-scale outcome can help with the design of optimized water exchange strategies which will depend on site-specific settings i.e. water volumes exchanged, the filtration rates, filter dimensions or filter medium properties. Therefore, biochar amendment provides the opportunity to enhance micropollutant retention in the filter medium for subsequent biodegradation.

**Table 2:** Linear sand-water partitioning coefficient K_s_ and apparent biochar-water partitioning coefficient K_bc_ fitted from field filtration experiment in comparison with values from the batch screening study.

	Fitted from filtration experiment	Batch study
Tetracycline		
K_s_ (L/kg)	1.4	n.a.
K_bc_ for CH (L/kg)	12.2	1859
K_bc_ for CH_CHI (L/kg)	12.7	648
Enrofloxacin		
K_s_ (L/kg)	<0.1	n.a.
K_bc_ for CH (L/kg)	7.0	1315
K_bc_ for CH_CHI (L/kg)	8.4	1551
Atrazine		
K_s_ (L/kg)	0.2	n.a.
K_bc_ for CH (L/kg)	24.6	292
K_bc_ for CH_CHI (L/kg)	<0.1	279
Diuron		
K_s_ (L/kg)	0.7	n.a.
K_bc_ for CH (L/kg)	19.2	2405
K_bc_ for CH_CHI (L/kg)	1.7	866

## 4 Conclusions

Each biochar was unique depending on its source of raw material, pyrolysis temperature, and surface characteristics, which resulted in distinct pharmaceuticals and pesticides removal from aqueous solution. The best micropollutant sorbent was CHE750 (benefit), however, its production requires sophisticated pyrolysis technology, which is not yet within reach of coastal aquaculture farmers in South Asia (cost). It may be desirable to use a blend of biochars as amendments in water filters or incorporate layers with different biochars, to optimize the retention of multiple pollutants typically found in real-world applications. Molecular dynamics simulations were broadly in agreement with our experimental results and indicated that the major sorption mechanism is via π-π stacking interactions. MD also confirmed the possibility of multilayer sorption for some of the micropollutants. Our study revealed that laboratory batch studies may overestimate the general sorption properties of the biochars by one or two orders of magnitude in comparison to practical, real-world applications. Nonetheless, the amendment of sand with biochar improved the retention of native micropollutants from brackish canal water in a realistic pilot-scale setting at a coastal aquaculture farm. This demonstrated the potential of this agricultural waste valorisation opportunity and for synergistic interactions between agriculture, biochar producers, and aquaculture in the Thai rural economy.

## Declaration of competing interest

The authors declare that they have no known competing financial interests or personal relationships that could have appeared to influence the work reported in this paper.

## Credit author statement

Wojciech Mrozik: Methodology, Investigation, Formal analysis, Data curation, Validation, Writing – original draft, Visualization. Babak Minofar: Software, Validation, Formal analysis, Data curation, Writing – review & editing. Thunchanok Thongsamer: Resources, Investigation, Writing – review & editing, Nathacha Wiriyaphong: Resources. Sasiwimol Khawkomol: Resources, Writing – review & editing. Jidapa Plaimart: Investigation, Writing – review & editing, John Vakros: Investigation, Writing – review & editing. Hrissi Karapanagioti: Writing – review & editing. Soydoa Vinitnantharat: Funding acquisition, Resources, Writing – review & editing. David Werner: Funding acquisition, Conceptualization, Methodology, Software, Validation, Writing – review & editing.
