# Supplementary material for: Valorisation of agricultural waste derived biochars in aquaculture to remove organic micropollutants from water – experimental study and molecular dynamics simulations
Source: J Environ Manage. 2021 Dec 15;300:113717. doi: 10.1016/j.jenvman.2021.113717 (PMC8542888; doi:10.1016/j.jenvman.2021.113717)
Supplement: Multimedia component 1 [file mmc1.docx]

*Supplementary Material*

**Performance of Agricultural Waste Derived Biochars in Aquaculture to Remove Organic Micropollutants from Water – Experimental Study and Molecular Dynamics Simulations**

| *Wojciech Mrozik^*1^, Babak Minofar^*2^, Thunchanok Thongsamer^3^, Nathacha Wiriyaphong^3^, Sasiwimol Khawkomol^3^, Jidapa Plaimart^1^, John Vakros^5^, Hrissi Karapanagioti^5^, Soydoa Vinitnantharat^3^ and David Werner^1^* |
| --- |

| *^1^School of Engineering, Newcastle University, Newcastle upon Tyne, NE1 7RU, United Kingdom*  *^2^Laboratory of Structural Biology and Bioinformatics, Institute of Microbiology of the Czech Academy of Sciences, Czech Republic*  *^3^Environmental Technology Program, School of Energy, Environment and Materials, King Mongkut’s University of Technology Thonburi, 126 Pracha-uthit road, Bangmod, Bangkok 10140, Thailand*  *^4^* *Energy and Environmental Engineering Center, Faculty of Engineering at Kamphaeng Saen, Kasetsart University, Nakhon Pathom, Thailand*  *^5^ Department of Chemistry, University of Patras, 26504 Patras, Greece*  *To whom correspondence should be addressed.  *e-mail:* [*wojciech.mrozik@ncl.ac.uk*](mailto:wojciech.mrozik@ncl.ac.uk)*, minofar@nh.cas.cz* |
| --- |

1. **Materials and Methods**

**Biochar preparation.** Biochar was produced from raw materials by the oil drum kiln method and in a pyrolysis reactor at the Center for Energy and Environmental Engineering, Kasetsart University, Kamphaeng Saen Campus, Thailand. Prior to carbonization, coconut husk was air-dried and weighed. The four types of agricultural residues (5 kg each) were carbonized in a 200 L oil drum kiln. The temperature profiles during combustion in the combustion chamber of the kiln were measured by using a thermocouple and are shown in Figure S1a. To start the carbonisation process, some prepared fuel (i.e. wood chips) was burnt in the front channel of the kiln. Next, the generated heat was passed into the kiln to help evaporate moisture from the raw batch. When the carbonization occurred (white smoke) the kiln was heated by internal circulation. After the smoke cleared, the front channel and whole outside surface of the kiln was covered by clay to prevent outside air from passing through. Then it was left for 12-24h for the complete carbonisation. After cooling, the biochar could be removed.

The pyrolysis reactor for biochar production in Thailand was set to 500 °C (maximum for this system) and biomass batch input was 5 kg. The chamber temperatures during combustion were monitored by a thermocouple (Figure S1b). The carbonization times were adjusted to outcomes from the drum kiln method. More detailed biochar characteristics and description can be found in our previous study (Khawkomol et al., 2021).

Biochar production in the UK was done by the UK Biochar Research Center at the University of Edinburgh at a fixed temperature of 550 ^o^C or 750 ^o^C. The pilot-scale (rotary kiln) unit was used for the coconut husk and rice straw biochar production because these two biomass materials did not readily pass through a bottleneck in the auger pyrolysis unit. A detailed description of the systems and method can be found in Masek et al. (Mašek et al., 2018). Due to limited feedstock availability, RSE750 could not be prepared, and CC could not be sent to the UK due to biosafety concerns as it was partially infested by fungi.


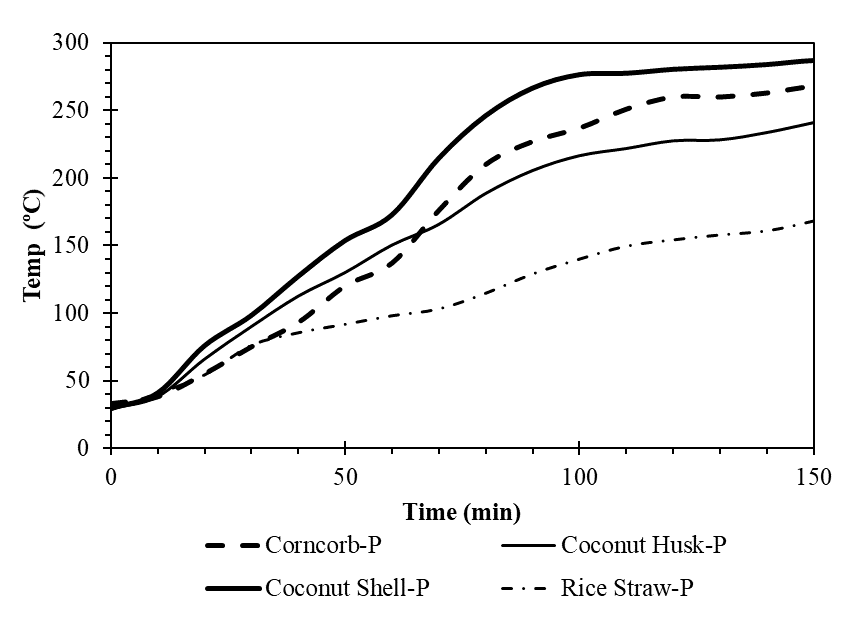

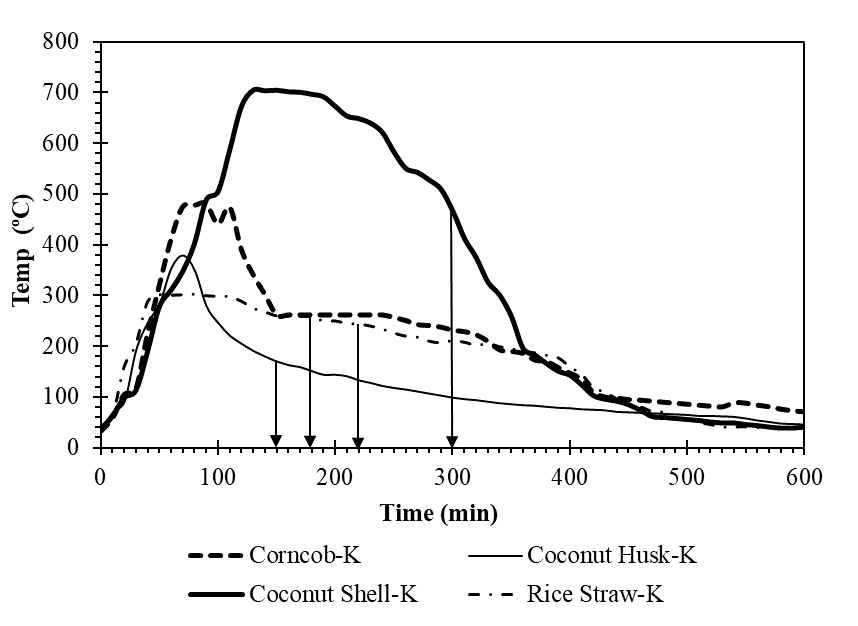
Figure S1. The temperature in the 200 L oil drum kiln and pyrolysis reactor (in Thailand) (a) oil drum kiln (b) pyrolysis reactor.

**Biochar properties.** The ultimate analysis was carried out to determine the percentage of carbon, hydrogen, nitrogen and sulfur elements using a Perkin Elmer 2400-II CHNS/O element analyser (Table S1).

Table S1. Chemical composition of raw materials (R) and biochars.

| Biomass |  | %C | %H | %N | %S | %O | O/C | H/C |
| --- | --- | --- | --- | --- | --- | --- | --- | --- |
| Corncob | R | 39.19 | 7.08 | 1.30 | 0.095 | 52.34 | 1.00 | 2.17 |
|  | CC_K | 60.36 | 3.03 | 1.81 | 0.12 | 34.68 | 0.43 | 0.60 |
| Coconut Husk | R | 46.02 | 5.74 | 0.04 | 0.05 | 48.15 | 0.78 | 1.50 |
|  | CH_K | 68.48 | 3.53 | 0.06 | 0.15 | 27.78 | 0.30 | 0.62 |
|  | CH_P_ | 61.97 | 4.32 | 0.8 | 0.07 | 32.84 | 0.40 | 0.84 |
|  | CHE550 | 59.04 | 1.4 | 0.82 | 0.47 | 38.27 | 0.49 | 0.28 |
|  | CHE750 | 56.73 | 0.93 | 1.39 | 0.51 | 40.44 | 0.53 | 0.20 |
| Coconut Shell | R | 51.30 | 6.80 | 0.40 | 0.04 | 41.46 | 0.61 | 1.59 |
|  | CS_K | 68.63 | 3.69 | 0.25 | 0.02 | 27.41 | 0.30 | 0.65 |
|  | CSE550 | 80.38 | 2.19 | 0.33 | 0.72 | 16.38 | 0.15 | 0.33 |
|  | CSE750 | 92.08 | 1.01 | 0.64 | 0.10 | 6.17 | 0.05 | 0.13 |
| Rice Straw | R | 37.04 | 5.90 | 1.01 | 0.16 | 55.89 | 1.13 | 1.91 |
|  | RS_K | 53.63 | 2.50 | 1.74 | 0.33 | 41.8 | 0.58 | 0.56 |
|  | RSE550 | 49.05 | 1.58 | 1.62 | 0.48 | 47.27 | 0.72 | 0.39 |

Note: R = Raw material K = Kiln

P= Pyrolysis in Thailand E = Pyrolysis in the UK

The surface area analysis was performed using a Tristar 3000 Micromeritics instrument that created nitrogen isotherms at -196^o^C. The samples were outgassed before measurement at 120 ^o^C under N_2_ flow for 120 min. The Specific Surface Area (SSA) was calculated using the Brunauer–Emmett–Teller (BET) equation, the microporous surface area using the t-plot method, and the pore size distribution using the Barrett-Joyner-Halenda (BJH) equation.

Skeletal density (g/mL) was obtained from the ratio of the dry mass of biochar (M_bc_) (g) to the skeletal volume of the biochar (V_bc,ske_) (mL). The biochar was boiled in water, then dried in an oven to obtain M_bc_, while V_bc,ske_ was obtained as the difference between the water volume of the boiled biochar/water slurry, and the total volume of this slurry.

The micro/mesoporosity was calculated from the BET isotherm.

The total pore volume was obtained from the difference between wet and dry weight of boiled biochar particles, externally dried with paper towels, while the skeletal volume was the ratio of the dry weight of biochar to its skeletal density. The total porosity was then calculated as the total pore volume relative to the total biochar volume.

Table S2. BET surface area (m^2^/g), skeletal density (g/cm^3^), micro/ mesopore porosity and total porosity of different biochars produced with the oil drum kiln method or 300/550/750 ˚C pyrolysis.

| **Biochar** | **BET surface area (m^2^/g)** | **Skeletal density (g/cm^3^)** | **Micro/Meso porosity** | **Total porosity** |
| --- | --- | --- | --- | --- |
| Corncob (CC) | n.d. | 1.68 | n.d. | 0.74 |
| Rice straw (RS) | 14.6 | 1.47 | 0.07 | 0.78 |
| RSE550 | 91 | 1.81 | 0.08 | >0.80 |
| Coconut husk (CH) | 11 | 1.41 | 0.04 | >0.80 |
| CHP | 5.3 | n/a | n/a | n/a |
| CHCHI (chitosan) | 0.4 | 3.27 | 0.01 | >0.80 |
| CHE550 | 5.4 | 1.59 | 0.01 | >0.80 |
| CHE750 | 272 | 2.30 | 0.25 | >0.80 |
| Coconut shell (CS) | n.d. | 1.53 | n.d. | 0.43 |
| CSE550 | 0.1 | 1.41 | 0.01 | 0.39 |
| CSE750 | n.d. | 1.54 | n.d. | 0.58 |

* n.d. = non-detectable, n/a – not available

As is evident in Table S2, CHE750 had the highest BET surface area measuring 272 m^2^/g, skeletal density measuring 2.30 g/cm^3^ and micro/meso-porosity measuring 0.25. The high surface area and microporosity of this CHE750 could thus explain the higher adsorption of the studied micropollutants. It is noticeable that CH biochar produced either by the oil drum kiln or fixed temperature pyrolysis had higher total porosity than the other biochars.

SEM analysis revealed that CC biochar had macropores of 10-20 um, whereas the rest of the biochars had macropores around 100 um (Figure S2).


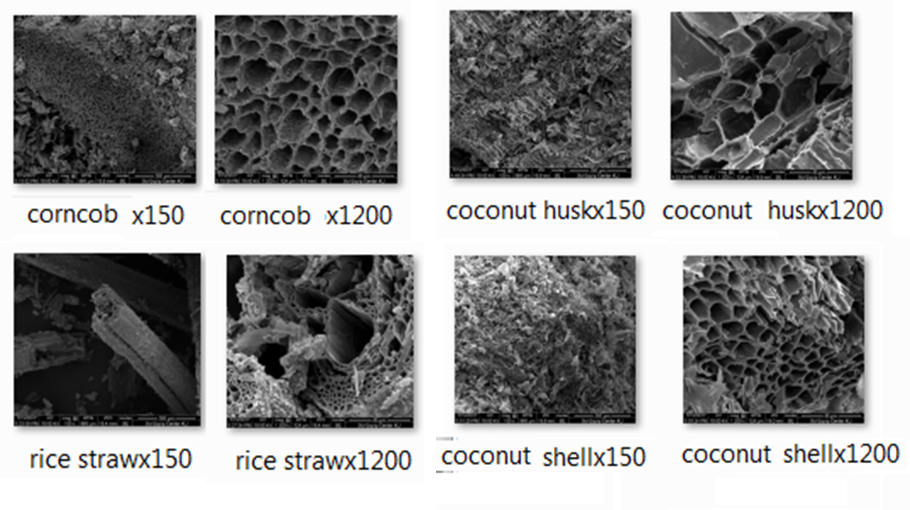
Figure S2. Selected SEM scans of biochars (oil drum method).

The biochars were grounded using a mortar and pestle and then sieved, and the <212 μm particle size fraction was used for the experiments.

**FTIR analysis.** The analysis were performed with a Nicolet 6700 spectrometer (Thermo Scientific). Prior to the analysis, biochar samples were mixed with KBr, then the mixture was grounded and formed into a tablet. The spectrum was scanned over the wavenumber ranging between 600 cm^-1^ to 4000 cm^-1^. Spectra are presented in Figure S3.


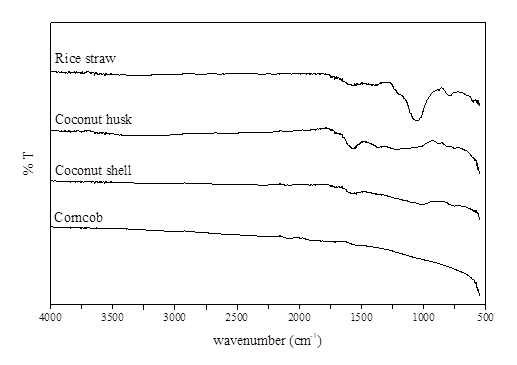

Figure S3. FTIR spectra of biochar produced in Thailand

CH, RS and CS biochars exhibited peaks in the region 900-675 cm^-1^ that are characteristic for aromatic substitution. Out of plane C-H bending bonds which are characteristic of the aromatic substitution pattern, existed for all biochars except a small peak in corncob biochar. For CC that pattern was very small. RS biochar showed broad bands at 1,050 and 787 cm^-1^. These are related to the stretching of C-O-C and Si-O-Si groups, respectively. The band intensity in the 1600 cm^-1^ region for all biochars (the smallest for CC) indicates conjugated C=C phenyl rings of ketones and quinones. That could be explained by the condensation of the biochar organic compounds (Collett et al., 2020; Domingues et al., 2017; Sarfaraz et al., 2020). CH also exhibited a small peak at 1,362 cm^-1^ which is indicative of lignin (C-O group of carboxyl and alcohol). FTIR revealed that all biochars consist of a high aromatic structure with attached carbonyl and hydroxyl groups. That indicates on the negative surface charge that RS is additionally enhanced by silica groups.

**Chromatographic analysis**. The selected micropollutants were analysed by a Thermo Fisher Ultimate 3000 UPLC system. It consisted of a binary pump, autosampler, column compartment and UV detector. The analysis was carried out at 230 nm wavelength. The column, ACE C-18 PFP column (2.1x100 mm, 1.7 um, HiChrom, Theale, UK), was thermostated at 40 ºC during the runs. The mobile phase consisted of solvent A (water with 0.1% formic acid) and B (acetonitrile with 0.1% formic acid). The flow rate was 0.4 mL/min. The gradient program was applied as follows: 0-0.25 min: 10% B; 0.25-9.5 min: 98% B; 9.5-10.5 min: 98% B; 10.5-11.5 min: 10% B; 11.5-14 min: 10% B (equilibration between samples). The injection was set at 80 μl. Figure S4 presents a chromatogram showing the resolution of all the micropollutants tested.


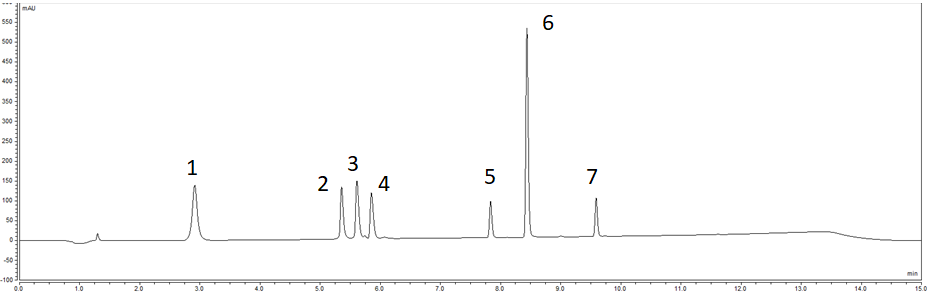


Figure S4. Chromatogram of micropollutants 1) acetaminophen, 2) oxytetracycline, 3) tetracycline, 4) enrofloxacin, 5) atrazine, 6) diuron and 7) diclofenac.

**Molecular Dynamics simulations.** For calculation of the partial charges of all molecules, ab initio geometry optimization using the Gaussian 03 package (Frisch et al., 2004) was performed by employing the B3LYP/6-31G* method. As we have used the general amber force field, which has different versions, we have chosen the GAFF force field from AmberTools18, which is part of the Amber 2018 program package. Quantum mechanical calculations yielded the optimized geometry of the molecules, and thereby, the atomic charges could be calculated from optimized geometries by application of the Restrained Electrostatic Potential (RESP) fitting scheme (Bayly et al., 1993) with the Antechamber program (Wang et al., 2006) which is a part of AmberTools18. To prepare the systems for studying the adsorption process, one molecule of each model biochar and 10 molecules of each organic micropollutant were solvated in different simulation boxes where the boxes were later solvated by water either without chitosan or NaCl, or with chitosan or NaCl. In this way, we could study the effect of co-adsorbent and ionic strength on the adsorption of organic micropollutants at the biochar surface. Different molecules were randomly distributed in the simulation boxes by using the Packmol package (Martínez and Martínez, 2003; Martínez et al., 2009). The simulation systems were minimized by steepest descent minimization to ensure the lack of unfavourable interactions in the systems. All systems later were equilibrated by 500 ps NVT (Canonical ensemble) restrained simulations followed by 500 ps NPT (isothermal–isobaric ensemble). A linear constraint solver (LINCS) algorithm (Hess et al., 1997) was employed for all bonds involving hydrogen atoms and short-range nonbonded interactions were truncated by cut off by 1.2 nm. Long-range electrostatic interactions were treated by the particle mesh Ewald method (Darden et al., 1993). Maxwell–Boltzmann distribution for production of initial velocities was used at 300 K. To maintain the temperature and the pressure of the systems constant during the simulations, the V-rescale coupling algorithm was used (Bussi et al., 2007) with the coupling constant of 0.1 ps. Production runs for data analysis were performed in the NPT ensemble for 100 ns at 300 K where a 2 fs time step was used. The Gromacs 4.6.5 program package was used for performing MD simulations (Berendsen et al., 1995; Lindahl et al., 2001; Van Der Spoel et al., 2005) while for visualizations and preparation of snapshots (Humphrey et al., 1996) Visual Molecular Dynamics (VMD) for LINUXAMD64, version 1.9.1 was used. For random distribution of molecules in the simulation boxes Packmol package of 2011 version has been used.

To quantify the interaction of biochar molecules with micro-pollutants molecules in aqueous solutions the General Amber Force Field (GAFF) model (Case et al., 2004) was used for all molecules including biochar models, micropollutants and chitosan molecules in MD simulations. For analyzing the MD data to reveal the adsorption of organic micropollutants at the surface of biochar molecules and explore the effect of salt and chitosan on the adsorption process the radial distribution function (RDF), g(r) was used. The radial distribution function describes the distribution of sorbate molecules around specific atoms or molecules in the system.


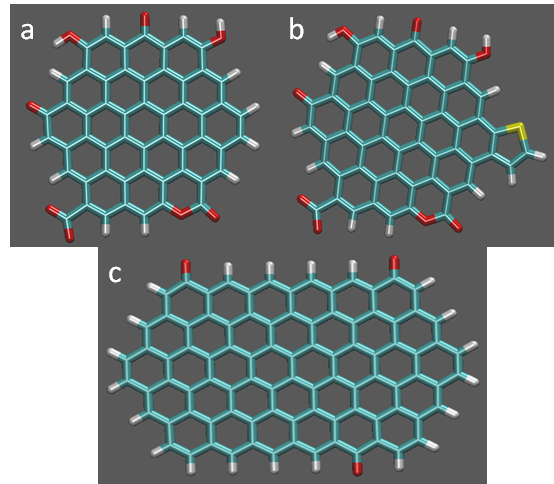


Figure S5: Chemical structures of model biochar molecules used in this study where molecule (a) has 18 benzene rings (R18), molecule (b) has 18 benzene rings with one thiophene group (R18S) and molecule (c) with 34 benzene rings (R34).

Table S3. Percentage of adsorption after 24h and 7 days batch experiment.

|  | Biochar type | CC | | RS | | RSE 550 | | CH | | CHP | | CHCHI | | CHE 550 | | CHE 750 | | CS | | CSE 550 | | CSE 750 | |
| --- | --- | --- | --- | --- | --- | --- | --- | --- | --- | --- | --- | --- | --- | --- | --- | --- | --- | --- | --- | --- | --- | --- | --- |
| Pollutant | BC loading  [mg/ L] | 24 h | 7 days | 24 h | 7 days | 24 h | 7 days | 24 h | 7 days | 24 h | 7 days | 24 h | 7 days | 24 h | 7 days | 24 h | 7 days | 24 h | 7 days | 24 h | 7 days | 24 h | 7 days |
| ACM | 100 | 2.9 | 3.1 | 3.5 | 3.7 | 9.6 | 9.9 | 8.8 | 9.1 | 8.9 | 9.2 | 0.0 | 0.2 | 2.0 | 2.2 | 64.0 | 65.0 | 4.9 | 5.2 | 1.8 | 2.0 | 11.0 | 11.5 |
|  | 200 | 3.7 | 3.9 | 4.1 | 4.4 | 12.2 | 12.5 | 10.7 | 11.0 | 10.8 | 11.1 | 2.1 | 2.3 | 3.0 | 3.3 | 76.5 | 77.5 | 7.0 | 7.3 | 2.7 | 3.0 | 18.4 | 18.8 |
|  | 500 | 7.0 | 7.3 | 5.9 | 6.2 | 21.4 | 21.8 | 18.5 | 18.8 | 18.7 | 19.0 | 7.7 | 7.9 | 11.9 | 12.2 | 100.0 | 100.0 | 14.9 | 15.1 | 4.2 | 4.5 | 36.9 | 37.4 |
|  | 1000 | 14.3 | 14.5 | 11.9 | 12.1 | 25.0 | 25.4 | 30.4 | 30.7 | 30.8 | 31.1 | 15.3 | 15.5 | 24.6 | 24.9 | 100.0 | 100.0 | 22.0 | 22.3 | 5.5 | 5.8 | 60.4 | 60.9 |
|  | 2000 | 18.1 | 18.3 | 18.4 | 18.7 | 50.4 | 50.8 | 57.3 | 57.6 | 58.0 | 58.3 | 31.9 | 32.1 | 39.4 | 39.7 | 100.0 | 100.0 | 38.2 | 38.5 | 6.9 | 7.1 | 96.9 | 97.3 |
|  | 5000 | 37.4 | 37.7 | 27.9 | 28.2 | 55.7 | 56.0 | 88.3 | 88.6 | 89.4 | 89.7 | 66.8 | 67.0 | 92.9 | 93.1 | 100.0 | 100.0 | 80.3 | 80.6 | 7.8 | 8.1 | 100.0 | 100.0 |
| OTC | 100 | 13.5 | 14.3 | 25.4 | 26.3 | 30.8 | 31.8 | 6.5 | 6.9 | 6.6 | 6.9 | 5.5 | 5.9 | 23.6 | 23.8 | 41.6 | 41.8 | 16.8 | 17.4 | 69.7 | 70.9 | 29.1 | 30.0 |
|  | 200 | 23.2 | 24.0 | 44.5 | 45.4 | 39.8 | 40.8 | 15.0 | 15.5 | 15.2 | 15.6 | 7.4 | 7.8 | 38.6 | 38.9 | 52.3 | 52.5 | 21.5 | 22.1 | 73.1 | 74.4 | 37.6 | 38.5 |
|  | 500 | 29.3 | 30.1 | 63.8 | 64.7 | 60.1 | 61.0 | 17.8 | 18.2 | 18.0 | 18.4 | 9.6 | 9.9 | 46.2 | 46.4 | 79.7 | 79.9 | 31.8 | 32.4 | 79.0 | 80.3 | 47.0 | 48.0 |
|  | 1000 | 50.4 | 51.2 | 87.4 | 88.3 | 65.1 | 66.0 | 24.0 | 24.4 | 24.3 | 24.7 | 17.4 | 17.7 | 70.2 | 70.5 | 95.8 | 96.0 | 37.0 | 37.6 | 80.9 | 82.1 | 59.1 | 60.0 |
|  | 2000 | 59.8 | 60.7 | 95.2 | 96.1 | 87.1 | 88.0 | 43.1 | 43.5 | 43.7 | 44.0 | 24.4 | 24.7 | 83.7 | 83.9 | 100.0 | 100.0 | 38.6 | 39.2 | 88.9 | 90.1 | 82.0 | 82.9 |
|  | 5000 | 87.0 | 87.8 | 99.2 | 100.0 | 89.7 | 90.7 | 78.6 | 79.0 | 79.6 | 80.0 | 27.9 | 28.3 | 96.8 | 97.0 | 100.0 | 100.0 | 83.8 | 84.4 | 92.8 | 94.0 | 97.4 | 98.3 |
| TC | 100 | 15.8 | 16.4 | 65.3 | 66.5 | 58.8 | 60.2 | 14.8 | 15.6 | 15.0 | 15.8 | 8.1 | 8.8 | 21.9 | 22.6 | 42.7 | 43.8 | 12.2 | 12.6 | 60.3 | 60.8 | 21.0 | 22.2 |
|  | 200 | 30.3 | 30.8 | 85.0 | 86.1 | 67.8 | 69.2 | 27.1 | 27.9 | 27.5 | 28.3 | 11.5 | 12.2 | 40.9 | 41.6 | 73.2 | 74.3 | 22.1 | 22.4 | 79.8 | 80.4 | 38.4 | 39.6 |
|  | 500 | 62.8 | 63.3 | 95.1 | 96.2 | 95.0 | 96.4 | 40.8 | 41.6 | 41.3 | 42.1 | 14.3 | 15.0 | 65.6 | 66.4 | 98.3 | 99.3 | 34.6 | 35.0 | 85.3 | 85.8 | 56.9 | 58.1 |
|  | 1000 | 77.2 | 77.7 | 96.3 | 97.5 | 96.4 | 97.8 | 56.7 | 57.5 | 57.4 | 58.2 | 24.1 | 24.8 | 94.1 | 94.9 | 99.5 | 100.0 | 40.1 | 40.5 | 89.6 | 90.2 | 81.9 | 83.1 |
|  | 2000 | 90.7 | 91.2 | 97.3 | 98.4 | 100.0 | 100.0 | 80.3 | 81.2 | 81.4 | 82.2 | 35.6 | 36.3 | 99.3 | 100.0 | 100.0 | 100.0 | 55.7 | 56.1 | 95.4 | 95.9 | 97.6 | 98.8 |
|  | 5000 | 99.2 | 99.7 | 98.5 | 99.6 | 100.0 | 100.0 | 98.8 | 99.6 | 100.1 | 100.0 | 39.9 | 40.6 | 100.0 | 100.0 | 100.0 | 100.0 | 85.5 | 85.8 | 97.7 | 98.2 | 100.0 | 100.0 |
| ENFL | 100 | 25.4 | 26.9 | 11.1 | 12.2 | 8.1 | 9.7 | 7.2 | 8.3 | 7.3 | 8.4 | 27.4 | 28.7 | 22.4 | 23.6 | 40.9 | 42.5 | 29.3 | 30.7 | 36.1 | 37.5 | 39.1 | 40.8 |
|  | 200 | 39.2 | 40.7 | 38.2 | 39.3 | 19.0 | 20.6 | 20.8 | 21.8 | 21.1 | 22.2 | 37.0 | 38.3 | 43.5 | 44.7 | 73.5 | 75.1 | 45.5 | 46.8 | 56.3 | 57.8 | 65.6 | 67.3 |
|  | 500 | 63.1 | 64.6 | 50.6 | 51.7 | 40.3 | 42.0 | 44.1 | 45.1 | 44.6 | 45.8 | 57.5 | 58.8 | 58.0 | 59.2 | 88.4 | 90.0 | 49.5 | 50.8 | 75.6 | 77.0 | 76.2 | 77.9 |
|  | 1000 | 78.2 | 79.7 | 63.5 | 64.6 | 51.6 | 53.2 | 57.3 | 58.3 | 58.1 | 59.2 | 76.4 | 77.8 | 84.2 | 85.4 | 100.0 | 100.0 | 57.3 | 58.7 | 82.4 | 83.9 | 83.9 | 85.6 |
|  | 2000 | 91.5 | 93.1 | 84.1 | 85.2 | 91.9 | 93.6 | 70.1 | 71.1 | 71.0 | 72.1 | 84.9 | 86.2 | 99.0 | 100.0 | 100.0 | 100.0 | 68.7 | 70.0 | 100.0 | 100.0 | 98.8 | 100.0 |
|  | 5000 | 98.9 | 100.4 | 87.6 | 88.7 | 96.3 | 98.0 | 87.6 | 88.7 | 88.8 | 89.9 | 100.0 | 100.0 | 100.0 | 100.0 | 100.0 | 100.0 | 85.7 | 87.1 | 100.0 | 100.0 | 100.0 | 100.0 |
| ATR | 100 | 0.6 | 1.1 | 5.2 | 6.1 | 14.1 | 15.1 | 2.5 | 2.9 | 2.5 | 2.9 | 2.9 | 3.3 | 9.0 | 9.2 | 48.0 | 48.3 | 3.5 | 4.1 | 7.1 | 8.3 | 11.8 | 12.7 |
|  | 200 | 2.2 | 2.7 | 10.4 | 11.3 | 19.3 | 20.3 | 5.5 | 6.0 | 5.6 | 6.0 | 5.3 | 5.6 | 13.2 | 13.5 | 72.8 | 73.0 | 7.3 | 7.9 | 12.8 | 14.0 | 16.4 | 17.3 |
|  | 500 | 3.3 | 3.8 | 18.1 | 19.0 | 30.5 | 31.4 | 12.5 | 12.9 | 12.7 | 13.1 | 11.2 | 11.5 | 19.7 | 20.0 | 95.5 | 95.8 | 13.5 | 14.1 | 15.9 | 17.1 | 24.7 | 25.6 |
|  | 1000 | 5.2 | 5.7 | 26.0 | 26.9 | 36.3 | 37.2 | 23.6 | 24.1 | 23.9 | 24.3 | 12.9 | 13.2 | 34.2 | 34.5 | 95.7 | 95.9 | 29.1 | 29.8 | 18.6 | 19.9 | 38.2 | 39.1 |
|  | 2000 | 17.1 | 17.6 | 31.0 | 31.9 | 74.4 | 75.4 | 36.4 | 36.9 | 36.9 | 37.3 | 20.2 | 20.6 | 47.4 | 47.6 | 95.8 | 96.1 | 50.0 | 50.7 | 24.8 | 26.0 | 75.2 | 76.1 |
|  | 5000 | 38.9 | 39.4 | 54.2 | 55.1 | 81.4 | 82.3 | 86.3 | 86.7 | 87.4 | 87.8 | 49.1 | 49.5 | 93.0 | 93.2 | 98.4 | 98.7 | 82.0 | 82.6 | 40.9 | 42.1 | 95.5 | 96.4 |
| DRN | 100 | 8.5 | 9.0 | 22.4 | 23.5 | 31.4 | 33.1 | 20.8 | 21.8 | 21.0 | 22.2 | 7.6 | 8.9 | 11.0 | 12.2 | 25.9 | 27.5 | 22.4 | 23.8 | 7.0 | 8.4 | 25.9 | 27.6 |
|  | 200 | 16.6 | 17.1 | 34.7 | 35.8 | 47.6 | 49.2 | 32.5 | 33.5 | 32.9 | 34.0 | 14.8 | 16.1 | 18.0 | 19.3 | 39.0 | 40.6 | 29.2 | 30.6 | 12.8 | 14.2 | 39.0 | 40.7 |
|  | 500 | 25.6 | 26.1 | 57.4 | 58.5 | 79.9 | 81.5 | 65.4 | 66.4 | 66.2 | 67.3 | 31.3 | 32.6 | 40.6 | 41.9 | 73.3 | 74.9 | 46.5 | 47.8 | 24.5 | 25.9 | 73.3 | 75.0 |
|  | 1000 | 52.4 | 52.9 | 65.4 | 66.5 | 98.7 | 100.0 | 87.0 | 88.0 | 88.2 | 89.3 | 47.8 | 49.1 | 71.9 | 73.1 | 96.8 | 98.4 | 71.5 | 72.9 | 39.3 | 40.7 | 96.8 | 98.5 |
|  | 2000 | 67.0 | 67.5 | 81.3 | 82.4 | 99.2 | 100.0 | 98.1 | 99.1 | 99.4 | 100.0 | 71.2 | 72.5 | 93.3 | 94.6 | 100.0 | 100.0 | 91.1 | 92.5 | 61.6 | 63.0 | 100.0 | 100.0 |
|  | 5000 | 94.9 | 95.4 | 95.5 | 96.6 | 100.0 | 100.0 | 100.0 | 100.0 | 100.0 | 100.0 | 94.9 | 96.2 | 100.0 | 100.0 | 100.0 | 100.0 | 99.2 | 100.0 | 88.5 | 89.9 | 100.0 | 100.0 |
| DIC | 100 | 4.7 | 4.9 | 0.9 | 1.1 | 3.1 | 3.4 | 0.3 | 0.6 | 0.3 | 0.6 | 15.2 | 15.4 | 3.2 | 3.4 | 11.9 | 12.9 | 3.2 | 3.5 | 2.2 | 2.4 | 9.0 | 9.4 |
|  | 200 | 6.9 | 7.2 | 8.0 | 8.2 | 6.2 | 6.5 | 1.0 | 1.3 | 1.1 | 1.4 | 27.7 | 27.9 | 8.4 | 8.6 | 27.5 | 28.5 | 7.2 | 7.4 | 3.0 | 3.2 | 13.9 | 14.3 |
|  | 500 | 8.6 | 8.8 | 9.9 | 10.1 | 8.6 | 8.9 | 5.3 | 5.6 | 5.3 | 5.6 | 31.8 | 32.0 | 14.5 | 14.7 | 58.2 | 59.2 | 14.0 | 14.3 | 3.9 | 4.2 | 19.1 | 19.5 |
|  | 1000 | 10.7 | 11.0 | 11.1 | 11.3 | 11.3 | 11.6 | 7.9 | 8.2 | 8.0 | 8.3 | 48.6 | 48.8 | 21.2 | 21.5 | 94.2 | 95.2 | 27.2 | 27.5 | 5.6 | 5.9 | 27.3 | 27.7 |
|  | 2000 | 15.9 | 16.1 | 13.7 | 13.9 | 20.0 | 20.3 | 9.7 | 10.0 | 9.9 | 10.2 | 54.2 | 54.4 | 28.7 | 28.9 | 100.0 | 100.0 | 36.0 | 36.3 | 6.1 | 6.4 | 41.7 | 42.1 |
|  | 5000 | 38.2 | 38.4 | 20.1 | 20.3 | 29.6 | 29.9 | 21.8 | 22.1 | 22.1 | 22.4 | 87.9 | 88.1 | 68.7 | 69.0 | 100.0 | 100.0 | 42.8 | 43.1 | 8.6 | 8.9 | 66.6 | 67.0 |

**Filtration field trial.** Figure S6 illustrates the experimental set-up of the water treatment systems to filter the canal water.


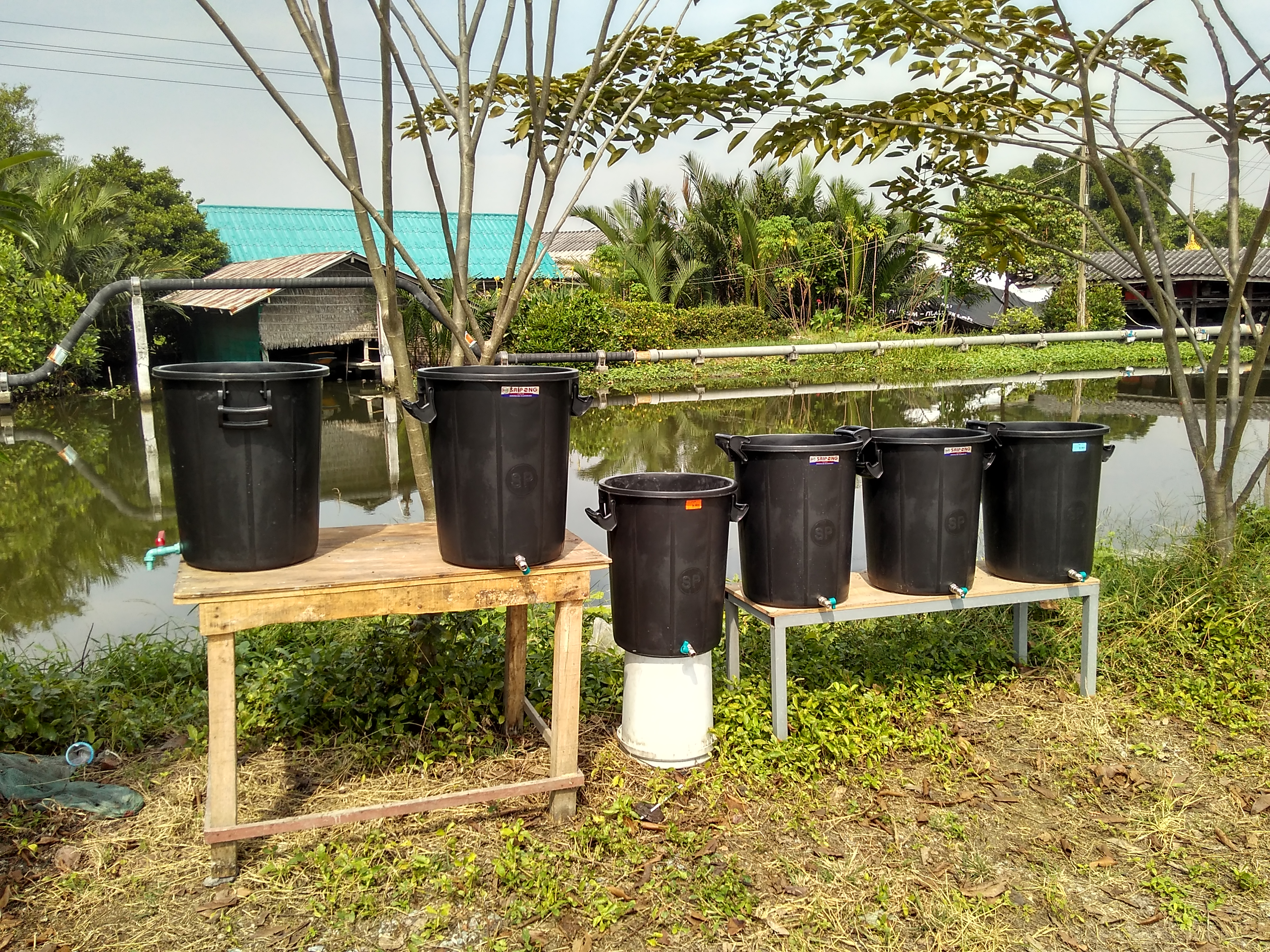


Figure S6. The field trial of biochar amendment benefits in canal water filtration

**Numerical simulation of the pilot-scale filtration experiment.** This model considered reversible sorption to sand, with or without first-order sorption rate kinetics, and reversible sorption to biochar, with or without sorption-retarded intraparticle diffusion of micropollutants in the biochar pore network to simulate intraparticle diffusion-based sorption kinetics. These models were implemented in Matlab (Version 2019a). The filter column experiments were simulated by considering pollutant transport by advection/dispersion retarded by sorption to the solid filter matrix. The pollutant distribution between water, and the solid matrix of the sand (S) and biochar (BC) particles was described by linear sorption coefficients, K_S_ and K_BC_, respectively. Parameters were expressed in SI units of moles, seconds, kilograms, and meters (Table S4). Sand-water (K_s_) and biochar-water sorption coefficients (K_bc_) were first fitted from the breakthrough data by assuming local sorption equilibrium and minimizing the deviation (sum of squared residuals) between the predicted and measured breakthrough curves for the control (sand) and biochar amended filters. The kinetic parameters (first-order sorption rate for the sand, and tortuosity factor for the intraparticle diffusion in biochar) were then fitted while keeping these K_bc_/K_s_ values constant to better account for the fronting and tailing observed in most of the measured breakthrough curves. The fitted sorption parameters from the filtration field trial were considered to provide a more realistic measure for the micropollutant retention by the biochar under field conditions as compared to those derived from batch tests.

Table S4: Independent and dependant filter column model variables and parameters and their dimensions

| t (s) | Time |
| --- | --- |
| x (m) | Distance from the column inlet |
| r (m) | Distance from the biochar particle centre |
| C_w_ (moles m^-3^) | Pollutant concentration in mobile water in between the S and BC particles |
| C_in_ (moles m^-3^) | Pollutant concentration in the influent |
| C_S_ (moles kg^-1^) | Pollutant concentration associated with the S particles |
| C_BC,ippw_ (moles m^-3^) | Pollutant concentration in intraparticle porewater of the BC particles |
| L_C_ (m) | Length of the column |
| R_C_ (m) | Radius of the column |
| Q_x_ (m^3^s^-1^) | Water flow through the column in the x-direction |
| v_x_ (ms^-1^) | Interstitial velocity of the mobile water in between the S and BC particles in the x-direction |
| D_aq_ (m^2^s^-1^) | Molecular diffusion coefficient of the pollutants in water |
| D_disp_ (m^2^s^-1^) | Dispersion coefficient for pollutants in mobile water in between the S and BC particles in the x-direction |
| M_S_ (kg) | Total dry mass of the S particles in the column |
| d_S_ (kg m^-3^) | Solid density of the S particles |
| K_S_ (m^3^kg^-1^) | Linear S particle solid-water partitioning coefficient for the pollutants |
| k_S_ (s^-1^) | First-order sorption kinetic rate, S particles |
| M_BC_ (kg) | Total dry mass of BC particles in the column |
| R_BC_ (m) | Biochar particle radius |
| p_BC_ (-) | BC intraparticle porosity |
| d_BC_ (kg m^-3^) | Solid density of the BC skeleton |
| τ_BC_ (-) | Tortuosity factor BC particles |
| K_BC_ (m^3^kg^-1^) | Linear BC solid-water partitioning coefficient for the pollutants |

Calculated filter column properties:

The volume fraction of the column filled with S particles, θ_S_, is defined as

$\theta_{S}=\frac{M_{S}}{d_{S}L_{C}\pi R_{C}^{2}}$ eq. 1

The volume fraction of the column filled with BC particles, θ_BC_, is defined as

$\theta_{BC}=\frac{M_{BC}}{{\left( 1-p_{BC} \right)d}_{BC}L_{C}\pi R_{C}^{2}}$ eq. 2

The volume fraction of the space in the column consisting of pores in between the S and BC particles, which is assumed to be entirely filled with mobile water, θ_w_, is defined as

$\theta_{w}=1-\theta_{S}-\theta_{BC}$ eq. 3

Differential equation for the local sorption equilibrium model:

The following partial differential equation governs the pollutant concentration in the mobile water phase moving in between the S and BC particles:

$\left( \theta_{w}+\theta_{S}d_{S}K_{S}+\theta_{BC}\cdot\left( p_{BC}+(1-p_{BC})d_{BC}K_{BC} \right) \right)\cdot\frac{d}{dt}C_{w}=\theta_{w}D_{disp}\frac{\partial^{2}}{\partial x^{2}}C_{w}-\theta_{w}v_{x}\frac{\partial}{\partial x}C_{w}$ eq. 4

Differential equations for the kinetic sorption model:

The following equations govern the pollutant concentration in the mobile water phase in between the S and BC particles:

$\theta_{w}\cdot\frac{d}{dt}C_{w}=\theta_{w}D_{disp}\frac{\partial^{2}}{\partial x^{2}}C_{w}-\theta_{w}v_{x}\frac{\partial}{\partial x}C_{w}-r_{ippwd,out}-r_{s}$ eq. 5

$r_{s}=-\theta_{w}k_{s}\left( \frac{C_{s}}{K_{s}}-C_{w} \right)$ eq. 6

$r_{ippwd,out}=\frac{\theta_{BC}}{\frac{4}{3}\pi R_{BC}^{3}}.4\pi R_{BC}^{2}.D_{eff,BC}.\frac{\partial}{\partial r}\left. C_{BC,ippw} \right|_{r=R_{BC}}$ eq. 7

$D_{eff,BC}=\frac{{p_{BC}.D}_{aq}}{\tau_{BC}}$ eq. 8

The following equation governs the pollutant concentration associated with the S particles

$\theta_{S}d_{S}\cdot\frac{d}{dt}C_{S}=r_{s}$ eq. 9

The following equations govern the pollutant concentration in the intraparticle porewater of BC particles

$\left( p_{BC}+\left( 1-p_{BC} \right)d_{BC}K_{BC} \right)\frac{d}{dt}C_{BC,ippw}=\frac{D_{eff,BC}}{r^{2}}.\frac{\partial}{\partial r}\left( r^{2}\frac{\partial}{\partial r}C_{BC,ippw} \right)$ eq. 10

Boundary conditions for the column:

The pollutant concentration at the column inlet is equal to the concentration in the column influent, C_in_,

$\left. C_{w} \right|_{x=0}=C_{in}$ eq. 11

A zero-concentration gradient boundary condition is enforced at the column outlet to assure there is no net dispersion flux across this boundary

$\left. \frac{\partial}{\partial x}C_{w} \right|_{x=L}=0$ eq. 12

To mimic the initial conditioning of the column with canal water for 15 min, the following changes in the flow conditions were simulated:

$v_{x}=\frac{Q_{x}}{\theta_{w}\pi R_{C}^{2}}$ for t = 0 to $\frac{\theta_{w}\pi R_{C}^{2}L_{C}}{Q_{x}}$ eq. 13

$v_{x}=0$ for t = $\frac{\theta_{w}\pi R_{C}^{2}L_{C}}{Q_{x}}$ to 900 s eq. 14

$v_{x}=\frac{Q_{x}}{\theta_{w}\pi R_{C}^{2}}$ for t > 900 s eq. 15

Boundary conditions for the BC particles in the kinetic sorption model:

In each column segment, the pollutant concentration in the mobile water phase is assumed to be equal to the pollutant concentration in BC intraparticle porewater at r = R_BC_.

$\left. C_{w} \right|_{x=x}=\left. C_{BC,ippw} \right|_{x=x, r=R_{BC}}$ eq. 16

Due to the assumed symmetry of the spherical BC particles, the pollutant concentration gradient is zero at r = 0.

$\left. \frac{\partial}{\partial r}C_{BC,ippw} \right|_{r=0}=0$ eq. 17

Table S5: Field study modelling parameters

| C_in_ (moles m^-3^) | Different for each pollutant (measured) |
| --- | --- |
| L_C_ (m) | 0.2 (measured) |
| R_C_ (m) | 0.135 (measured) |
| Q_x_ (m^3^s^-1^) | 1.67*10^-5^ (measured) |
| D_aq_ (m^2^s^-1^) | 2.7 *10^-8^/MW^0.71^ (estimated, MW = molecular weight of the pollutant in g/mol) |
| D_disp_ (m^2^s^-1^) | 4*10^-8^ (estimated based on (Werner et al., 2012)) |
| M_S_ (kg) | 10 (measured, 11 for the control with S only) |
| d_S_ (kg m^-3^) | 2500 (estimated based on the density of sand particle (Bushnaf et al., 2017)) |
| K_S_ (m^3^kg^-1^) | Fitted from control with S only |
| k_S_ (s^-1^) | Fitted from control with S only |
| M_BC_ (kg) | 1 (measured, 0 for the control with S only) |
| R_BC_ (m) | 0.000077 (estimated from sieve size <212 μm) |
| p_BC_ (-) | 0.8 (from Table S1) |
| d_BC_ (kg m^-3^) | 1410 (from Table S1) |
| τ_BC_ (-) | Fitted from S & BC filters |
| K_BC_ (m^3^kg^-1^) | Fitted from S & BC filters |

Sensitivity analysis:

As shown in Table S6, the fitted sorption coefficients K_BC_ derived from the field trial experiments were not sensitive to uncertainty in any of the estimated parameter values (D_disp_, d_S_, R_BC_ from Table S5). For the parameter values derived from the control experiments (K_S_, k_S_), the best fit K_BC_ value of diuron for CHCHI was sensitive to changes in the best-fit value of its sand-water partitioning coefficient K_S_, with a +10% change in the K_S_ value resulting in a -35.3% change in the best-fit K_BC_ value. This sensitivity existed because the difference between the K_S_ and K_BC_ parameter values was relatively small in this instance, or, in other words, the relative contribution of sorption to the sand to the overall diuron sorption was not negligible in the column amended with CHCHI biochar. To a lesser extent such sensitivity also existed for tetracycline in the biochar amended columns (Table S6). The best-fit K_BC_ values were not sensitive to uncertainty in the first-order sorption kinetic rates k_S_ derived from the control experiments.

Table S6: Sensitivity analysis. Percent change in the best-fit linear BC solid-water partitioning coefficients K_BC_ for +10% change in the estimated model input parameter values (D_disp_, d_S_, R_BC_), or for +10% change in the input parameter values derived from the control experiments (K_S_, k_S_).

| Pollutant and biochar amendment in column | Percent change in K_BC_ for +10% change in D_disp_ | Percent change in K_BC_ for +10% change in  d_S_ | Percent change in K_BC_ for +10% change in R_BC_ | Percent change in K_BC_ for +10% change in K_s_ | Percent change in K_BC_ for +10% change in k_S_ |
| --- | --- | --- | --- | --- | --- |
| TC, CH | 0% | -1.6% | 0% | -13.1% | 0% |
| TC, CHCHI | 0% | 0.8% | 0% | -11.0% | 0% |
| ENFL, CH | 0% | 0% | 0% | 0% | 0% |
| ENFL, CHCHI | 0% | 0% | 0% | 0% | 0% |
| ATR, CH | 0% | 0.4% | 0% | -0.8% | 0% |
| ATR, CHCHI | 0% | 0% | 0% | 0% | 0% |
| DRN, CH | 0% | 0% | 0% | -4.2% | 0% |
| DRN, CHCHI | 0% | 0% | 0% | -35.3% | 0% |

1. **Results and Discussion**

Table S7. Parameters of applied isotherms for selected biochars

|  |  | Linear | | | Langmuir | | | | BET | | | | | Freundlich | | | |
| --- | --- | --- | --- | --- | --- | --- | --- | --- | --- | --- | --- | --- | --- | --- | --- | --- | --- |
| Sorbent | Sorbate | K [L/kg] | SSR [mg/kg]^2 | R2 | C_max_ [mg/kg] | K_L_ [L/mg] | SSR [mg/kg]^2 | R2 | Cs [mg/L] | q_m_ [mg/kg] | B | SSR [mg/kg]^2 | R2 | K_fr_ [mg/kg][L/mg]^(1/n) | 1/n | SSR [mg/kg]^2 | R2 |
| CC | OTC | 1268 | 254955 | 0.773 | 30960 | 0.041 | 264299 | 0.764 | 1.10 | 317 | 4.41 | 91038 | 0.919 | 1757 | 2.038 | 127042 | 0.887 |
| CC | TC | 2206 | 429459 | 0.740 | 2347 | 2.592 | 43756 | 0.974 | 15.65 | 2087 | 47.41 | 44971 | 0.973 | 1798 | 0.508 | 46804 | 0.972 |
| CC | ENFL | 3370 | 66699 | 0.984 | 21354 | 0.175 | 61546 | 0.985 | 1.27 | 1160 | 7.67 | 22197 | 0.995 | 3187 | 0.893 | 49447 | 0.988 |
| CC | DRN | 914 | 61889 | 0.820 | 3040 | 0.392 | 57599 | 0.832 | 1.48 | 306 | 39.58 | 31547 | 0.908 | 844 | 0.719 | 50525 | 0.853 |
| RS | OTC | 3675 | 363966 | 0.918 | 5420 | 1.152 | 207813 | 0.953 | 1.48 | 1385 | 13.97 | 125902 | 0.972 | 3060 | 0.659 | 123325 | 0.972 |
| RS | TC | 20688 | 2596736 | 0.917 | 13614 | 2.742 | 587624 | 0.981 | 1.81 | 8124 | 8.47 | 705844 | 0.977 | 15059 | 0.752 | 1144345 | 0.963 |
| RS | ENFL | 1879 | 898874 | 0.526 | 2937 | 1.131 | 708131 | 0.627 | 15.8 | 2499 | 21.40 | 715668 | 0.623 | 1651 | 0.700 | 777395 | 0.590 |
| RS | DRN | 2685 | 112974 | 0.965 | 46147 | 0.061 | 129871 | 0.959 | 1.66 | 1948 | 1.84 | 47657 | 0.985 | 3038 | 1.255 | 53171 | 0.983 |
| CH | ACM | 651 | 142428 | 0.551 | 10777 | 0.063 | 144440 | 0.545 | 1.04 | 96 | 174.88 | 58085 | 0.817 | 757 | 1.765 | 136730 | 0.569 |
| CH | TC | 1668 | 114389 | 0.915 | 19122 | 0.092 | 124607 | 0.908 | 1.17 | 430 | 57.84 | 76815 | 0.943 | 1806 | 1.216 | 101799 | 0.925 |
| CH | ENFL | 1130 | 198844 | 0.624 | 1792 | 1.172 | 122036 | 0.769 | 456 | 2032 | 431.62 | 123558 | 0.766 | 1005 | 0.660 | 141825 | 0.732 |
| CH | ATR | 288 | 19152 | -1.28 | 264 | 11.913 | 3915 | 0.533 | 2.41 | 157 | 181.03 | 1933 | 0.769 | 251 | 0.226 | 2982 | 0.644 |
| CH | DRN | 2717 | 644572 | 0.583 | 2387 | 4.385 | 187126 | 0.879 | 1.50 | 967 | 74.32 | 28054 | 0.982 | 2100 | 0.417 | 64768 | 0.958 |
| CS | OTC | 1186 | 959273 | 0.452 | 32263 | 0.037 | 971495 | 0.445 | 0.98 | 445 | 0.24 | 56658 | 0.968 | 4680 | 5.720 | 61304 | 0.965 |
| CS | TC | 1164 | 227332 | 0.764 | 19459 | 0.062 | 240143 | 0.751 | 1.40 | 872 | 0.75 | 63723 | 0.934 | 1720 | 2.266 | 61948 | 0.936 |
| CS | ATR | 383 | 17156 | 0.358 | 457 | 2.688 | 4822 | 0.820 | 2.34 | 214 | 28.37 | 3518 | 0.868 | 342 | 0.449 | 3959 | 0.852 |
| CS | DRN | 2380 | 417328 | 0.850 | 31718 | 0.078 | 431043 | 0.845 | 0.96 | 419 | 127.48 | 38161 | 0.986 | 2518 | 1.136 | 411761 | 0.852 |
| CHCHI | DRN | 879 | 35395 | 0.860 | 1523 | 1.038 | 16797 | 0.934 | 1.73 | 381 | 23.27 | 3745 | 0.985 | 783 | 0.597 | 7156 | 0.972 |
| CHCHI | DIC | 1423 | 544419 | 0.669 | 26695 | 0.055 | 562203 | 0.658 | 1.28 | 958 | 0.66 | 229309 | 0.861 | 2408 | 2.532 | 218005 | 0.867 |
| CHE550 | ACM | 240 | 44154 | -6.21 | 208 | 122.702 | 5807 | 0.051 | 1005 | 223 | 24413.59 | 8126 | -0.32 | 207 | 0.031 | 5921 | 0.033 |
| CHE550 | OTC | 2761 | 464376 | 0.877 | 29037 | 0.101 | 499795 | 0.867 | 1.11 | 857 | 3.93 | 284654 | 0.924 | 3398 | 1.438 | 356061 | 0.905 |
| CHE550 | TC | 3168 | 956364 | 0.538 | 2208 | 11.112 | 374536 | 0.819 | 1.53 | 1128 | 157.50 | 78929 | 0.962 | 2307 | 0.343 | 112323 | 0.946 |
| CHE550 | ENFL | 3197 | 550731 | 0.779 | 5406 | 0.942 | 442923 | 0.822 | 1.31 | 974 | 104.87 | 349003 | 0.860 | 2598 | 0.590 | 361868 | 0.855 |
| CHE550 | ATR | 706 | 150587 | 0.602 | 13095 | 0.056 | 154638 | 0.591 | 1.03 | 111 | 95.08 | 17158 | 0.955 | 1070 | 3.045 | 77891 | 0.794 |
| CHE550 | DRN | 1281 | 299712 | -0.37 | 1063 | 9.513 | 37890 | 0.826 | 26.1 | 1048 | 230.18 | 36217 | 0.834 | 1029 | 0.303 | 20632 | 0.905 |
| CHE750 | ATR | 10387 | 3373768 | 0.804 | 6974 | 4.168 | 1050682 | 0.939 | 2.72 | 4841 | 17.86 | 1059591 | 0.938 | 7400 | 0.609 | 1273918 | 0.926 |
| CSE750 | ACM | 1219 | 220910 | 0.094 | 893 | 31.584 | 125412 | 0.486 | 1.52 | 456 | 358.92 | 9914 | 0.959 | 959 | 0.260 | 73868 | 0.697 |
| CSE750 | OTC | 2951 | 1458423 | 0.732 | 55990 | 0.054 | 1503193 | 0.723 | 0.81 | 401 | 12.68 | 108705 | 0.980 | 8958 | 3.311 | 221766 | 0.959 |
| CSE750 | TC | 2856 | 328145 | 0.831 | 4915 | 0.932 | 246899 | 0.873 | 1.33 | 901 | 50.02 | 121547 | 0.937 | 2367 | 0.603 | 165323 | 0.915 |
| CSE750 | ENFL | 7021 | 1149631 | 0.874 | 18378 | 0.469 | 1016216 | 0.889 | 4.03 | 10857 | 2.81 | 1106327 | 0.879 | 6348 | 0.871 | 1063140 | 0.884 |
| CSE750 | ATR | 978 | 247159 | 0.623 | 9833 | 0.107 | 253792 | 0.613 | 1.01 | 154 | 119.61 | 45598 | 0.930 | 1729 | 3.700 | 203834 | 0.689 |
| RSE550 | OTC | 3578 | 842286 | 0.861 | 68385 | 0.053 | 890013 | 0.854 | 0.89 | 750 | 3.49 | 100146 | 0.984 | 5794 | 1.866 | 248615 | 0.959 |
| RSE550 | ENFL | 1089 | 227752 | 0.427 | 916 | 8.748 | 81003 | 0.796 | 2.49 | 583 | 50.54 | 62427 | 0.843 | 899 | 0.377 | 67431 | 0.830 |
| RSE550 | DRN | 4763 | 1510224 | 0.665 | 3548 | 5.513 | 948755 | 0.790 | 1.22 | 1391 | 138.09 | 50943 | 0.989 | 3278 | 0.362 | 286271 | 0.937 |
| CSE550 | TC | 15295 | 1666779 | 0.938 | 108673 | 0.147 | 1726813 | 0.936 | 2.93 | 27184 | 1.56 | 1642582 | 0.939 | 17188 | 1.096 | 1547555 | 0.942 |
| CSE550 | DRN | 716 | 15649 | 0.920 | 5719 | 0.139 | 15819 | 0.919 | 1.38 | 236 | 22.6 | 683 | 0.997 | 690 | 0.853 | 13892 | 0.929 |


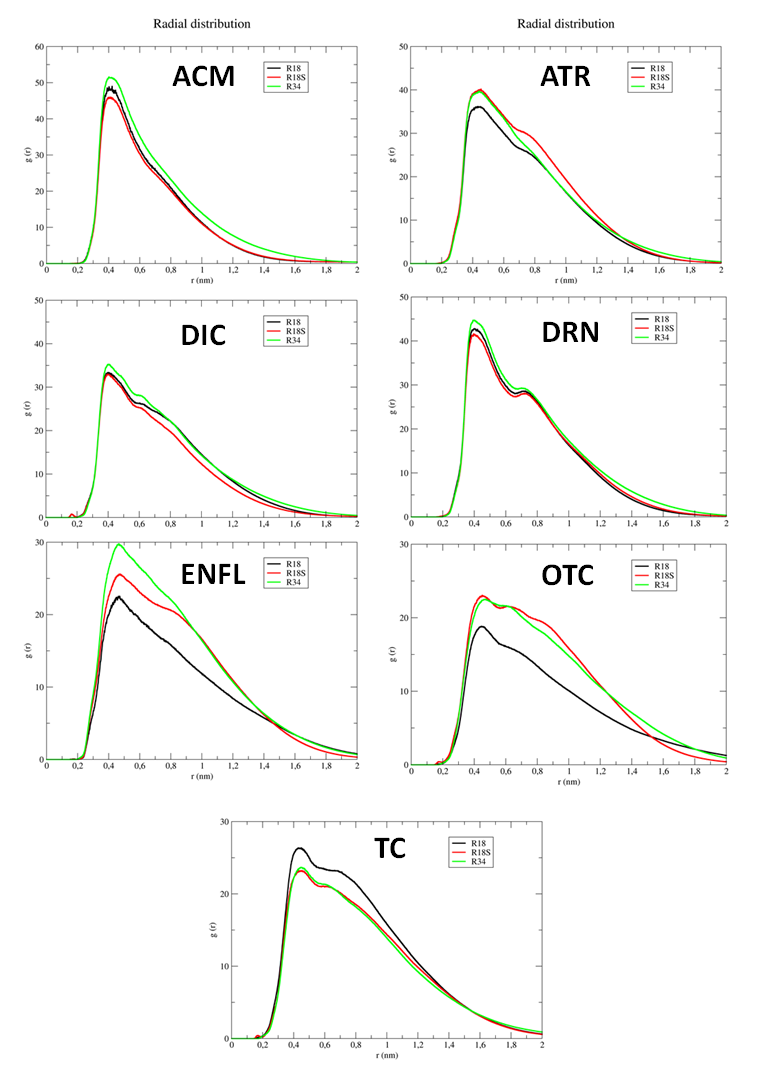


Figure S7. Radial distribution function of the center of mass of micropollutant molecules around R18, R18S and R34 biochar molecules.


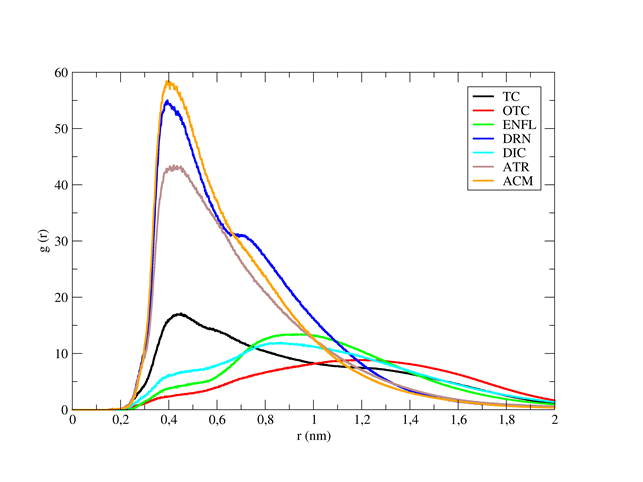
 Figure S8. The radial distribution function of the centre of mass of organic micropollutant molecules around an R18 biochar molecule in a mixture of micropollutants in solution with 1M NaCl

1. **References**

Bayly, C.I., Cieplak, P., Cornell, W., Kollman, P.A., 1993. A well-behaved electrostatic potential based method using charge restraints for deriving atomic charges: the RESP model. The Journal of Physical Chemistry 97, 10269-10280.

Berendsen, H.J.C., van der Spoel, D., van Drunen, R., 1995. GROMACS: A message-passing parallel molecular dynamics implementation. Comput. Phys. Commun. 91, 43-56.

Bushnaf, K.M., Mangse, G., Meynet, P., Davenport, R.J., Cirpka, O.A., Werner, D., 2017. Mechanisms of distinct activated carbon and biochar amendment effects on petroleum vapour biofiltration in soil. Environmental Science: Processes & Impacts 19, 1260-1269.

Bussi, G., Donadio, D., Parrinello, M., 2007. Canonical sampling through velocity rescaling. The Journal of Chemical Physics 126, 014101.

Case, D.A., Darden, T.A., Cheatham, T.E.I., Simmerling, C.L., Wang, J., Duke, R.E., Luo, R., Merz, K.M., Wang, B., Pearlman, D.A., Crowley, M., Brozell, S., Tsui, V., Gohlke, H., Mongan, J., Hornak, V., Cui, G., Beroza, P., Schafmeister, C., Caldwell, J.W., Ross, W.R., Kollman, P.A., 2004. AMBER 8. University of California, San Francisco.

Collett, C., Mašek, O., Razali, N., McGregor, J., 2020. Influence of Biochar Composition and Source Material on Catalytic Performance: The Carboxylation of Glycerol with CO2 as a Case Study. Catalysts 10, 1067.

Darden, T., York, D., Pedersen, L., 1993. Particle mesh Ewald: An N⋅log(N) method for Ewald sums in large systems. The Journal of Chemical Physics 98, 10089-10092.

Domingues, R.R., Trugilho, P.F., Silva, C.A., Melo, I.C.N.A.d., Melo, L.C.A., Magriotis, Z.M., Sánchez-Monedero, M.A., 2017. Properties of biochar derived from wood and high-nutrient biomasses with the aim of agronomic and environmental benefits. PLOS ONE 12, e0176884.

Frisch, M.J., Trucks, G.W., Schlegel, H.B., Scuseria, G.E., Robb, M.A., Cheeseman, J.R., Montgomery, J.J.A., Vreven, T., Kudin, K.N., Burant, J.C., Millam, J.M., Iyengar, S.S., Tomasi, J., Barone, V., Mennucci, B., Cossi, M., Scalmani, G., Rega, N., Petersson, G.A., Nakatsuji, H., Hada, M., Ehara, M., Toyota, K., Fukuda, R., Hasegawa, J., Ishida, M., Nakajima, T., Honda, Y., Kitao, O., Nakai, H., Klene, M., Li, X., Knox, J.E., Hratchian, H.P., Cross, J.B., Bakken, V., Adamo, C., Jaramillo, J., Gomperts, R., Stratmann, R.E., Yazyev, O., Austin, A.J., Cammi, R., Pomelli, C., Ochterski, J.W., Ayala, P.Y., Morokuma, K., Voth, G.A., Salvador, P., Dannenberg, J.J., Zakrzewski, V.G., Dapprich, S., Daniels, A.D., Strain, M.C., Farkas, O., Malick, D.K., Rabuck, A.D., Raghavachari, K., Foresman, J.B., Ortiz, J.V., Cui, Q., Baboul, A.G., Clifford, S., Cioslowski, J., Stefanov, B.B., Liu, G., Liashenko, A., Piskorz, P., Komaromi, I., Martin, R.L., Fox, D.J., Keith, T., Al-Laham, M.A., Peng, C.Y., Nanayakkara, A., Challacombe, M., Gill, P.M.W., Johnson, B., Chen, W., Wong, M.W., Gonzalez, C., Pople, J.A., 2004. Gaussian 03, Revision 03. Gaussian, Inc., Wallingford, CT.

Hess, B., Bekker, H., Berendsen, H.J.C., Fraaije, J.G.E.M., 1997. LINCS: A linear constraint solver for molecular simulations. J. Comput. Chem. 18, 1463-1472.

Humphrey, W., Dalke, A., Schulten, K., 1996. VMD: Visual molecular dynamics. Journal of Molecular Graphics 14, 33-38.

Khawkomol, S., Neamchan, R., Thongsamer, T., Vinitnantharat, S., Panpradit, B., Sohsalam, P., Werner, D., Mrozik, W., 2021. Potential of biochar derived from agricultural residues for sus-tainable management. Sustainability 13.

Lindahl, E., Hess, B., van der Spoel, D., 2001. GROMACS 3.0: a package for molecular simulation and trajectory analysis. Molecular modeling annual 7, 306-317.

Martínez, J.M., Martínez, L., 2003. Packing optimization for automated generation of complex system's initial configurations for molecular dynamics and docking. J. Comput. Chem. 24, 819-825.

Martínez, L., Andrade, R., Birgin, E.G., Martínez, J.M., 2009. PACKMOL: A package for building initial configurations for molecular dynamics simulations. J. Comput. Chem. 30, 2157-2164.

Mašek, O., Buss, W., Roy-Poirier, A., Lowe, W., Peters, C., Brownsort, P., Mignard, D., Pritchard, C., Sohi, S., 2018. Consistency of biochar properties over time and production scales: A characterisation of standard materials. J. Anal. Appl. Pyrolysis 132, 200-210.

Sarfaraz, Q., Silva, L., Drescher, G., Zafar, M., Severo, F., Kokkonen, A., Molin, G., Shafi, M., Shafique, Q., Solaiman, Z., 2020. Characterization and carbon mineralization of biochars produced from different animal manures and plant residues. Scientific Reports 10, 955.

Van Der Spoel, D., Lindahl, E., Hess, B., Groenhof, G., Mark, A.E., Berendsen, H.J.C., 2005. GROMACS: Fast, flexible, and free. J. Comput. Chem. 26, 1701-1718.

Wang, J., Wang, W., Kollman, P.A., Case, D.A., 2006. Automatic atom type and bond type perception in molecular mechanical calculations. J. Mol. Graphics Modell. 25, 247-260.

Werner, D., Karapanagioti, H.K., Sabatini, D.A., 2012. Assessing the effect of grain-scale sorption rate limitations on the fate of hydrophobic organic groundwater pollutants. J. Contam. Hydrol. 129-130, 70-79.
